# Ocean warming and acidification alter the behavioral response to flow of the sea urchin *Paracentrotus lividus*

**DOI:** 10.1002/ece3.5678

**Published:** 2019-10-17

**Authors:** Mishal Cohen‐Rengifo, Antonio Agüera, Tjeerd Bouma, Saloua M'Zoudi, Patrick Flammang, Philippe Dubois

**Affiliations:** ^1^ Laboratoire de Biologie des Organismes Marins et Biomimétisme Institut de recherches en Biosciences Université de Mons Mons Belgium; ^2^ Laboratoire de Biologie Marine (CP160/15) Université Libre de Bruxelles Brussels Belgium; ^3^ Institute of Marine Research Austevoll Research Station Storebø Norway; ^4^ Department of Estuarine and Delta Systems Royal Netherlands Institute for Sea Research (NIOZ) Utrecht University Yerseke The Netherlands

**Keywords:** behavior, biomechanics, climate change, flow, hydrodynamics, ocean acidification, ocean warming, physiology, sea urchin

## Abstract

Ocean warming (OW) and acidification (OA) are intensively investigated as they pose major threats to marine organism. However, little effort is dedicated to another collateral climate change stressor, the increased frequency, and intensity of storm events, here referred to as intensified hydrodynamics. A 2‐month experiment was performed to identify how OW and OA (temperature: 21°C; pH_T_: 7.7, 7.4; control: 17°C‐pH_T_7.9) affect the resistance to hydrodynamics in the sea urchin *Paracentrotus lividus* using an integrative approach that includes physiology, biomechanics, and behavior. Biomechanics was studied under both no‐flow condition at the tube foot (TF) scale and flow condition at the individual scale. For the former, TF disk adhesive properties (attachment strength, tenacity) and TF stem mechanical properties (breaking force, extensibility, tensile strength, stiffness, toughness) were evaluated. For the latter, resistance to flow was addressed as the flow velocity at which individuals detached. Under near‐ and far‐future OW and OA, individuals fully balanced their acid‐base status, but skeletal growth was halved. TF adhesive properties were not affected by treatments. Compared to the control, mechanical properties were in general improved under pH_T_7.7 while in the extreme treatment (21°C‐pH_T_7.4) breaking force was diminished. Three behavioral strategies were implemented by sea urchins and acted together to cope with flow: improving TF attachment, streamlining, and escaping. Behavioral responses varied according to treatment and flow velocity. For instance, individuals at 21°C‐pH_T_7.4 increased the density of attached TF at slow flows or controlled TF detachment at fast flows to compensate for weakened TF mechanical properties. They also showed an absence of streamlining favoring an escaping behavior as they ventured in a riskier faster movement at slow flows. At faster flows, the effects of OW and OA were detrimental causing earlier dislodgment. These plastic behaviors reflect a potential scope for acclimation in the field, where this species already experiences diel temperature and pH fluctuations.

## Graphical abstract

The capacity of sea urchins to resist hydrodynamics, through a physiological, biomechanical, and behavioral integrative approach was evaluated. Sea urchins fully balance their acid‐base status under the five climate change treatments but skeleton growth was halved reflecting a change in resource allocation. Mechanical properties of tube feet showed no clear trend and seem to have a less relevant role in attachment capacity compared to the interaction of behavioral traits. Streamlined behavior was severely affected in the extreme treatment, but compensated by increasing the number of attached tube feet and by moving faster to avoid the flow. This set of behavioral traits together with physiological and mechanical responses further determines attachment capacity.

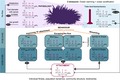

## 1 INTRODUCTION

Over the past ~300 million years of Earth's history, several elevated atmospheric CO_2_ events have been reported, but nowadays, CO_2_ is been released at unprecedented fast rates due to anthropogenic activity (Honisch et al., 2012). Indeed, atmospheric CO_2_ concentration increased from preindustrial levels of 280 ppm to the current value of 410 ppm (Dlugokencky & Tans, 2018) and might rise to approximately 1,000 ppm by the end of this century (Caldeira & Wickett, 2003; IPCC, 2014). Consequently, sea‐surface temperature has increased by approximately 0.8°C in the past 150 years and is predicted to rise by a further 2–4.5°C by the end of this century (IPCC, 2014). Simultaneously, the ocean has absorbed ~26% of anthropogenic atmospheric CO_2_ (Le Quéré, Takahashi, Buitenhuis, Rödenbeck, & Sutherland, 2010), inducing changes in the carbonate system equilibrium such as reduced carbonate ion concentration and pH. These processes are merged under the term ocean acidification (OA, Feely et al., 2009). Seawater pH is predicted to decrease by 0.3–0.4 units by 2,100 and by a further 0.7 by 2,300 according to the RCP 8.5 scenario (Caldeira & Wickett, 2003; IPCC, 2014; Orr et al., 2005).

The effects of predicted ocean warming (OW) and/or OA on marine invertebrates range from individual physiologies (Pörtner, 2010; Somero, 2002), to changes in population dynamics (Ling, Johnson, Ridgway, Hobday, & Haddon, 2009), food availability (Hoegh‐Guldberg & Pearse, 1995; O'Connor, Piehler, Leech, Anton, & Bruno, 2009), increased diseases (Lester, Tobin, & Behrens, 2007), or mortality (Coma et al., 2009). OA increases the energetic cost of building calcified skeletons (Bach, 2015; Pörtner, 2008) and could influence dissolution of existing skeletons if they are not protected by organic layers (Dery, Collard, & Dubois, 2017; Manno, Sandrini, Tositti, & Accornero, 2007; Melzner et al., 2011). In addition, significant interactive effects of OW and OA have been observed on fertilization and early development, survival, calcification, or growth (e.g., Byrne, 2011; Kroeker et al., 2013; Kroeker, Kordas, Crim, & Singh, 2010).

Ocean warming and acidification take often the leading role in discussions about how global climate change will alter marine biota, while little attention has been dedicated to another collateral stressor, here referred to as intensified hydrodynamics. There is strong evidence suggesting that the frequency and intensity of extratropical cyclones in the North Atlantic basin have increased since the 1950s (Hartmann et al., 2013). As cyclones get their energy from warm water (Gautam, Cervone, Singh, & Kafatos, 2005), it is suggested that the recent increase in storminess is in nexus with human‐induced global warming (Donat et al., 2011; Komar, 2007; Latif, Keenlyside, & Bader, 2007), though there is no absolute consensus about this relationship (IPCC, 2013; Ulbrich, Leckebusch, & Pinto, 2009). In addition, increased storminess can intensify the severity of wind‐driven waves. A rise of the yearly mean wave height in the North East Atlantic by 20% to 40% has been already observed during the 20th century (Bacon & Carter, 1991; Bertin, Prouteau, & Letetrel, 2013).

As wave‐induced water motion can potentially dislodge organisms from the substratum, hydrodynamics is considered as a major driver shaping the benthic intertidal and upper infralittoral communities (Denny, 1988). To resist dislodgement caused by hydrodynamic forces, benthic organisms rely on both the mechanics of their adhesive organs and their behavior (Hofmann & Todgham, 2010). If OW and OA affect the ability of benthic organisms to withstand the hydrodynamic stress, the structure and dynamics of the ecosystems where they play key roles can be significantly affected (Agüera, Koppel, Jansen, Smaal, & Bouma, 2015; Britton‐Simmons, Foley, & Okamoto, 2009; Duggins, 1981).

The reported effects of low pH on biomechanics of noncalcified materials include no significant effect on TF mechanical properties in the starfish *Asterias rubens*, reduced mechanical performance of the byssus in bivalves, decreased clapping force in the scallop *Pecten maximus*, and lowered spore attachment in intertidal rhodophyta algae (Collard, Catarino, Bonnet, Flammang, & Dubois, 2013; George & Carrington, 2018; Guenther, Miklasz, Carrington, & Martone, 2017; Li, Liu, Zhan, Xie, & Zhang, 2017; O'Donnell, George, & Carrington, 2013; Schalkhausser et al., 2013). Behavioral studies under climate change conditions mainly concentrate on OA effects on fishes (Cripps, Munday, & McCormick, 2011; Dixson, Munday, & Jones, 2010; Domenici, Allan, McCormick, & Munday, 2012; Ferrari et al., 2012; Hamilton, Holcombe, & Tresguerres, 2014; Jutfelt, Bresolin de Souza, Vuylsteke, & Sturve, 2013; Munday et al., 2009; Nilsson et al., 2012; Simpson et al., 2011) and, to a lesser extent, on marine invertebrates focusing on predator–prey relationships (Bibby, Cleall‐Harding, Rundle, Widdicombe, & Spicer, 2007; Chan, Grünbaum, Arnberg, & Dupont, 2016; Dodd, Grabowski, Piehler, Westfield, & Ries, 2015; Manríquez et al., 2013).

In adult echinoids, the effects of simultaneous OW and OA vary according to the stressors magnitude, acclimation period, species, and response variable (e.g., Dubois, 2014; Kroeker et al., 2010; Wittmann & Pörtner, 2013). Regarding physiology, metabolism upregulation resulted from both warming and acidification in *Heliocidaris erythrogramma* (Carey, Harianto, & Byrne, 2016), while in *Paracentrotus lividus*, it resulted only from acidification (Catarino, Bauwens, & Dubois, 2012). However, a longer exposure to these stressors can follow acclimation as observed in *Sterechinus neumayeri* (Morley, Suckling, Clark, Cross, & Peck, 2016; Suckling et al., 2015). Concerning behavior, simultaneous OW and OA increased grazing activity in *Amblypneustes pallidus*, while in *H. erythrogramma*, feeding rate increased with warming (Burnell, Russell, Irving, & Connell, 2013; Carey et al., 2016). After long exposure to OW and OA, *Loxechinus albus* feeding preference disappeared in response to acidification and not warming, while the vertical foraging speed and tenacity were not affected (Manríquez et al., 2017).

The impact of hydrodynamics on mechanical and behavioral responses of echinoids have been extensively studied. High flow velocity or wave exposure reduced movement distance and speed, feeding rate and particle capture efficiency, influenced righting and spine streamlining behavior, and enhanced TF mechanical properties (Cohen‐Rengifo et al., 2018; Cohen‐Rengifo, Moureaux, Dubois, & Flammang, 2017; Dance, 1987; Denny & Gaylord, 1996; George & Carrington, 2014; Jacinto & Cruz, 2012; Kawamata, 1998; Lauzon‐Guay, Scheibling, & Barbeau, 2006; Lissner, 1980; Morse & Hunt, 2013; Stewart & Britton‐Simmons, 2011; Tuya, Cisneros‐Aguirre, Ortega‐Borges, & Haroun, 2007). However, mechanical resistance and behavioral responses of sea urchins under the simultaneous impact of OW and OA coupled to an additional hydrodynamic stress have never been investigated. As echinoids play a major structuring role in many coastal ecosystems through their grazing activity results in a reference (Steneck, 2013), the impact of OW and OA in a more hydrodynamic ocean could be of tremendous importance.

**Figure 1:**
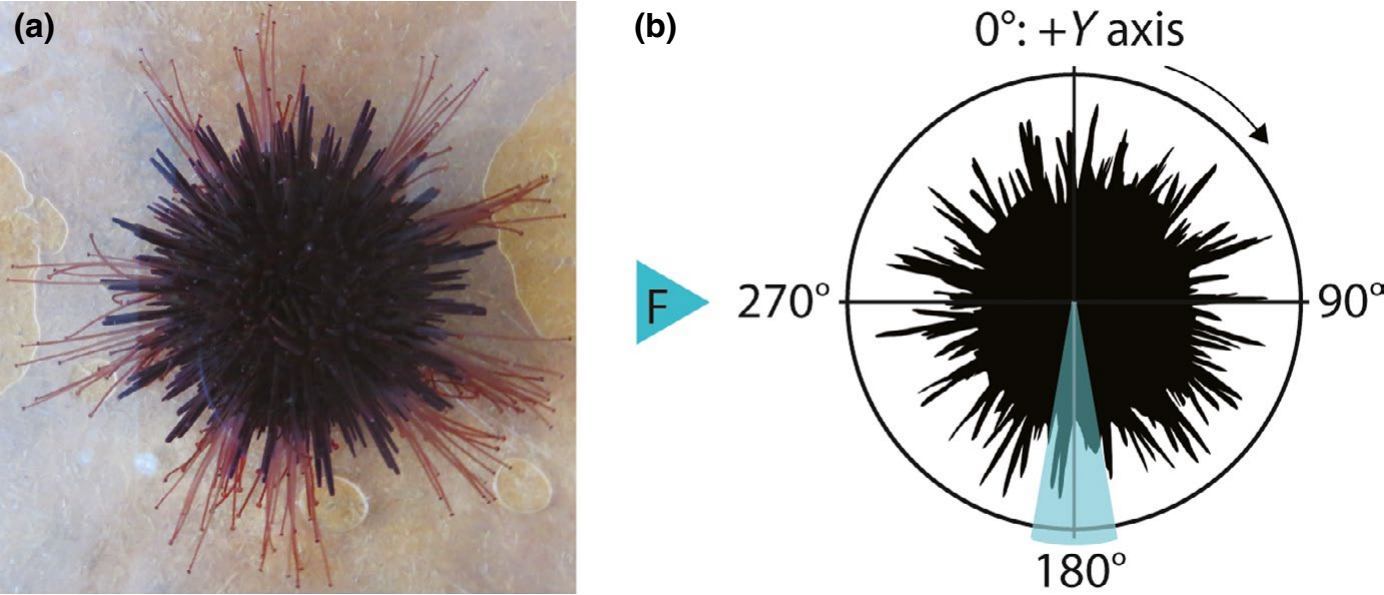
(a) Sea urchin *Paracentrotus lividus* showing its extended tube feet. (b) Unit circle illustrating the zone between 180 and 10° where spines angle was measured with respect of the positive *Y* mathematical axis that corresponds to 0°. F: flow direction

The present study seeks to understand how OW and OA influence the resistance to an increasing flow regime in the echinoid *P. lividus* (Figure 1a). During a two‐month experiment, echinoids were exposed to 6 fully crossed treatments including two temperatures (17 and 21°C) and three pH_T_ (7.9, 7.7, and 7.4) according to the RCP8.5 scenario (IPCC, 2014). Potential functional effects on TF adhesive and mechanical properties under no‐flow conditions, as well as behavioral responses associated with movement and shape modification (through spine reorientation) under flow conditions, were evaluated to identify their role in the resistance to dislodgment. Compensation of extracellular pH, respiration rate, and somatic growth was also studied to address the overall physiological state.

## 2 MATERIALS AND METHODS

### 2.1 Sea urchin collection and experimental setup

In September 2014, 216 sea urchins (ambital test diameter, *d*_test_ = 17.1–34.4 mm) were hand‐collected from intertidal pools in Aber beach (48°14′15″N 4°27′18″W), France. Animals were transported alive to Belgium and let to acclimate for 12 days (Cohen‐Rengifo et al., 2018). At day 3, individuals were soaked during 24 hr in the fluorescent marker calcein (20 mg/L) to tag their skeletal components (Rodríguez, Hernéndez, & Clemente, 2016; Russell & Urbaniak, 2004).

Aquaria were held within climate rooms to allow constant seawater temperature (°C). Between 10 and 20% of the water volume within each tank was renewed each day. Once a day, temperature, salinity, pH in NIST scale (pH_NIST_), and electromotive force (mV) were measured as in Cohen‐Rengifo et al. (2018). Daily electromotive force measurements were converted to pH in total scale (pH_T_) using Tris/AMP buffer calibration (DelValls & Dickson, 1998). At day 5 of acclimation, temperature and pH were progressively modified from control values (17°C‐pH_T_7.9, +0.5°C/day, −0.05 pH per day). Target treatments (17°C‐pH_T_7.7, 17°C‐pH_T_7.4, 21°C‐pH_T_7.9, 21°C‐pH_T_7.7, 21°C‐pH_T_7.4) were reached after 12 days and were maintained for 12 more weeks. A computer‐controlled IKS system was employed to manage experimental pH by bubbling CO_2_ independently into each aquarium. IKS‐pH measurements were calibrated against Metrohm pH meter daily measurements. Treatments were triplicated independently (1 aquarium = 1 replicate, 6 treatments = 18 aquaria).

To determine seawater total alkalinity (*A*_T‐SW_), a 50 ml sample was collected once a week from each aquarium and immediately filtered (0.22 μm MilliPore) and fixed with HgCl_2_ 7% (w/vol). Potentiometric titrations according to Gran (1950) were carried out using a Titrino 718 STAT (Metrohm AG). Quality control was performed using reference material supplied by Dickson laboratory (University of California, Batch 135), with measures being always within ±2% of the reference value. pCO_2_ and the concentration of the carbonate system components (CO_2_, ${\text{HCO}}_{3}^{-}$, ${\text{CO}}_{3}^{2-}$) and calcite and aragonite saturation states (Ω) were calculated using the software CO_2_SYS (Pierrot, Lewis, & Wallace, 2006) and the dissociation constants for carbonate from Mehrbach, Culberson, Hawley, and Pytkowicz (1973) refitted by Dickson and Millero (1987).

In each of the 18 aquaria, 12 sea urchins were separated into three compartments (plastic‐mesh cages). One compartment contained three individuals that were employed to monitor physiological state. Another compartment housed three individuals employed to evaluate biomechanics under no‐flow conditions, and the last compartment housed six individuals employed to evaluate skeletal growth as well as biomechanics and behavior under hydrodynamic conditions. Samplings were carried out at week 1 (w1, just after the 12‐day acclimation), 8 (w8), and 12 (w12). Individuals were fed ad libitum with Zeigler Bros., Inc. (USA) food pellets.

### 2.2 Physiological state

Physiological state was assessed by evaluation of growth, respiration rate (µmol O_2_ hr^−1 ^g^−1^), coelomic fluid (CF) pH (pH_NIST‐CF_), total alkalinity (*A*_T‐CF_, mmol/kg_sw_), and buffer capacity. CF buffer capacity was calculated as the difference between total alkalinity of the CF and that of the seawater (Δ*A*_T‐CF_ = *A*_T‐CF_–*A*_T‐seawater_, mmol/kg_sw_). Measurements were taken at w1 and w8 on three individuals per aquarium (*n* = 3). Only growth was measured at w1 and w12 in 6 individuals per aquarium (*n* = 3), considering the increment (%) in ambital sea urchin test diameter with spines (*d*_urchin_), test diameter (*d*_test_) and height (*h*_test_) without spines, ambital spine length (*l*_spine_), and the increment (mm) in jaw size. See S2 in Appendix S1 for details.

### 2.3 Microhabitat conditions

At Aber beach, sea urchins inhabiting intertidal pools are mainly wedged in self‐burrowed pits where water movement and gas exchange are reduced during low tide. We hypothesized that sea urchins could be locally preadapted to low pH at the scale of the microhabitat. Therefore, pH_T_ and A_T_ over, below, and inside (i.e., the CF) an echinoid within its pit (Figure S1) were measured in two tide pools at different tidal periods. See S3 in Appendix S1 for details.

### 2.4 No‐flow biomechanics

Biomechanical variables were measured at w1 and w8 following Cohen‐Rengifo et al. (2018). Tenacity tests were performed on a whole individual or on a single tube foot disk. An individual was clamped with a metal grab and let to attach to a glass aquarium, while a single tube foot was directly let to attach to a glass piece. The metal grab or the glass piece was connected to an Instron 5543 force stand (© Illinois Tool Works Inc). A force perpendicular to the substratum was applied at a constant speed of 25 mm/min. Force and displacement were recorded at a frequency of 10 Hz until detachment. Sea urchin detachment force (*F*_urchin_, N) or tube foot disk detachment force (*F*_disk_, N) was documented. The adhesive surface area of a single tube foot disk (*S*_disk_, mm^2^) was estimated from stained footprints (Cohen‐Rengifo et al., 2017; Santos & Flammang, 2007) while that of the sea urchin (*S*_urchin_) was calculated by multiplying *S*_disk_ and the number of adoral TF (Cohen‐Rengifo et al., 2018). *F*_urchin_ was measured for three individuals per aquarium while *F*_disk_ and *S*_disk_ for three TF per sea urchin per aquarium (1 aquarium = 1 replica, *n* = 3). Tenacity of the sea urchin (*T*_urchin,_ MPa) and disk (*T*_disk_, MPa) was calculated as the respective detachment force per unit of adhesive surface area.

Traction tests were performed on single tube foot stem that was clipped and connected to the same instrument. A pulling force perpendicular to the test was applied at a constant speed of 25 mm/min until stem breakage. Breaking force (N) was recorded. Transverse histological sections of 3 stems per individual per aquarium (*n* = 3) were prepared to measure the cross‐sectional surface area of the stem connective tissue layer (*S*_CT_, μm^2^), as this tissue bears all the external load exerted on a tube foot (Santos & Flammang, 2005). *S*_CT_ was then used to calculate stem mechanical properties such as tensile strength (MPa), extensibility (unitless), stiffness (MPa), and toughness (MJ/m^3^).

### 2.5 Biomechanics and behavior under hydrodynamic conditions

#### 2.5.1 Flume tank setup and sea urchin dislodgement

At week 10, the whole experimental setup together with 107 alive sea urchins (18 aquaria, each containing 6 individuals; 1 died) was transported (duration 90 min) to the Royal Netherlands Institute for Sea Research (NIOZ‐Yerseke). Hydrodynamic trials were performed in a recirculating flume tank (maximal speed: 90 cm/s; working section: 0.6 m wide × 2 m length) that was calibrated with a Vectrino Acoustic Doppler Velocimeter (Nortek Group). Before the trials, individuals were acclimated to their respective treatments for one week. In the aquaria, temperature, pH_T‐SW_, and salinity were measured and controlled daily as described above. Hydrodynamic trials lasted 14 days (*n* = 14). In the flume tank, seawater parameters (mean ± *SD*) were salinity = 31.6 ± 0.5 (*n* = 14) and pH_T‐SW_ = 7.90 ± 0.01 (*n* = 14), while temperature was measured during the first 7 days for the control = 17.0 ± 0.4°C (*n* = 7) and during the last 7 days for the high‐temperature treatment = 20.9 ± 0.5°C (*n* = 7). Because individuals were maintained for less than 30 min in the flume tank, pH_T‐SW_ was not manipulated.

A unidirectional flow parallel to the substratum was generated (Bouma et al., 2005). A transparent polymethyl methacrylate plate was employed as attachment substratum. Flow velocity (*V*_F_, in cm/s) in the flume tank was set at 30 cm/s to create an abrupt transition of water motion conditions. Each individual (6 per aquarium, *n* = 3) was placed alone, oral‐side down in the middle of the working section under a plastic basket to limit displacement and allow attachment. After 5 min, the basket was removed, and *V*_F_ was increased by 5 cm/s every 2 min (flow2′ regime) until reaching 90 cm/s or until detachment. During a hydrodynamic trial, detachment velocity (*V*_Det_, cm/s) and behavioral variables (see below) were measured for each individual. Afterward, skeletons were dried at 50°C for 48 hr and cleaned with NaOCl 2.5% for 2 hr. Four additional hours were needed to clean Aristotle's lanterns.

#### 2.5.2 Active movement

Sea urchin active movement velocity (*V*_Mov_, cm/min) and direction (Dir_Mov_, degrees, circular variable) under flow conditions were measured following Cohen‐Rengifo et al. (2018). Briefly, individuals were photographed from above to track their position, and pictures were analyzed with the ImageJ v1.50i software MTrackJ plug‐in. Sea urchin coordinates per picture were extracted with the software R v3.4.1 (R Development Core Team, 2015) and used to estimate *V*_Mov_ and Dir_Mov_. To determine at which *V*_F_, *V*_Mov_ is significantly different from zero, a 95% confidence interval was calculated with a confidence level of 95% (*α* = 0.05).

#### 2.5.3 Spine orientation and shape analysis

Sea urchin planform silhouettes were photographed with a Canon Powershot SX260HS camera at every *V*_F_ to measure spine angle (Spine°, degrees, circular variable). A single spine situated between 180 ± 10° (considering that 0° corresponds to the positive *Y* mathematical axis; Figure 1b) was chosen for each individual. Using ImageJ v1.50i software, the angle formed by the tip of the spine with respect to 0° was measured in a clockwise direction (Cohen‐Rengifo et al., 2018).

To determine whether the streamlined shape under climate change conditions was adopted earlier than in the control (i.e., 55 cm/s), planform shape was analyzed using pictures taken between 30–55 cm/s and at *V*_Det_. Shape analyses were performed with MATLAB v2015 software Image Processing Toolbox™, based on shape indices (S1 in Appendix S1) and elliptic Fourier coefficients (Agüera & Brophy, 2011; Cohen‐Rengifo et al., 2018). Briefly, twenty elliptic Fourier harmonics were calculated per individual and *V*_F_. Shape of individuals from the same *V*_F_ was averaged, so that a reconstructed overall shape can be visualized per *V*_F_ according to treatment.

#### 2.5.4 Attached tube feet

A waterproof Canon Powershot d10 camera was placed under the transparent working section and took 10 frames/min of individuals' oral side. Individuals moved very fast reaching areas where photographs could not be taken. Therefore, a flow velocity gradient in which velocity increased every minute (Flow1′ regime) was implemented. Sea urchin photographs were used to count the number of attached TF was counted. The density of total attached TF relative to oral test surface area (TF_att_, mm^−2^) and the percentage of total attached TF relative to the number of adoral TF (TF_att_%, %) were calculated. The number of adoral TF (from the ambitus toward the oral peristome) was estimated on cleaned tests, considering that a pair of ambulacral pores corresponds to a single tube foot (Santos & Flammang, 2007; Smith, 1978).

### 2.6 Statistics

Statistical tests are explained in detail in S4 in Appendix S1. A first general linear model (model 1) was developed to determine the probability of dislodgement according to flow velocity (*V*_F_), morphology (*d*_test_, *h*_test_, *l*_spine_), and flow regime (Flow2′, Flow1′) between treatments. A second model (model 2) was conceived to identify the behavioral variables controlling *V*_Det_, while a third model (model 3) aimed to determine whether *V*_Mov_ varied according to treatment and/or *V*_F_. See Tables S1–S3 in Appendix S1 for model details. Model selection and validation were performed using R v3.4.1 software (R Development Core Team, 2015) as in Cohen‐Rengifo et al. (2018).

## 3 RESULTS

Target treatments were maintained stable during the 81 days of experiment. Seawater and carbonate system parameters per treatment, averaged over the whole experiment, are available in Table 1. Values for seawater parameters and all linear variables are throughout expressed as mean ± *SD*.

**Table 1:** Seawater and carbonate system parameters (mean ± *SD*, *n* = 81 except for *A*_T_: *n* = 12) averaged over 12 weeks of experiment

Nominal treatment	Effective temperature (°C)	Effective pH_T‐SW_ (pH units)	Salinity (PSU)	*A*_T‐SW_ (mmol/kg_sw_)	*ρ*CO_2_ (μatm)	CO_2_ (μmol/kg_sw_)	${\text{HCO}}_{3}^{-}$ (μmol kg_sw_^−1^)	${\text{CO}}_{3}^{2-}$ (μmol/kg_sw_)	ΩCa	ΩAr
17°C‐pH_T_7.9	17.3 ± 0.3	7.90 ± 0.06	31.8 ± 0.01	2.31 ± 0.34	607 ± 97	22 ± 3	2,035 ± 280	126 ± 31	3.1 ± 0.8	2.0 ± 0.5
17°C‐pH_T_7.7	17.4 ± 0.3	7.69 ± 0.07	32.1 ± 0.04	2.18 ± 0.30	961 ± 223	34 ± 8	1,980 ± 266	76 ± 18	1.9 ± 0.4	1.2 ± 0.3
17°C‐pH_T_7.4	17.7 ± 0.3	7.42 ± 0.09	32.1 ± 0.03	2.17 ± 0.37	1817 ± 415	64 ± 14	2,057 ± 332	45 ± 16	1.1 ± 0.4	0.7 ± 0.3
21°C‐pH_T_7.9	21.1 ± 0.2	7.85 ± 0.06	31.9 ± 0.01	2.20 ± 0.27	661 ± 96	21 ± 3	1,921 ± 214	122 ± 27	3.0 ± 0.7	1.9 ± 0.4
21°C‐pH_T_7.7	20.9 ± 0.7	7.67 ± 0.09	32.1 ± 0.03	2.10 ± 0.37	1,019 ± 395	33 ± 1	1,895 ± 341	79 ± 2	1.9 ± 0.5	1.3 ± 0.3
21°C‐pH_T_7.4	21.4 ± 0.7	7.44 ± 0.10	32.1 ± 0.04	2.05 ± 0.44	1695 ± 451	53 ± 14	1,926 ± 385	51 ± 22	1.2 ± 0.5	0.8 ± 0.4

Note

*A*_T‐SW_, total alkalinity, calcite (ΩCa), and aragonite (ΩAr) saturation state.

### 3.1 Physiological state

Increment in echinoid diameter, with and without spines, showed the highest values in control conditions (21 ± 6% and 19 ± 3%, respectively) and the lowest ones in the extreme treatment (10 ± 5% and 2 ± 3%, respectively). However, these differences were only significant for sea urchin diameter with spines (*p* = .001, Table 2). Jaw size increment of control individuals significantly doubled that of individuals in all other treatments (*p* < .001).

**Table 2:** Means values (±*SD*, *n* = 3) for physiological parameters in *Paracentrotus lividus* according to experimental treatment at w1 and w8

	Time	Treatments						ANOVA	
		17°C‐pH_T_7.9 control	17°C‐pH_T_7.7	17°C‐pH_T_7.4	21°C‐pH_T_7.9	21°C‐pH_T_7.7	21°C‐pH_T_7.4 extreme	*F*_5,12_	*p*‐Value
Respiration rate (O^2^ µmol hr^−1^g^−1^)	w1	0.72 ± 0.24	0.82 ± 0.20	0.81 ± 0.22	0.89 ± 0.16	0.95 ± 0.15	0.86 ± 0.15	1.234*	.357
	w8	0.88 ± 0.22	1.08 ± 0.23	1.03 ± 0.36	1.28 ± 0.39	1.17 ± 0.17	1.02 ± 0.15	2.111	.134
pH_NIST‐CF _(pH units)	w1	7.61 ± 0.08	7.56 ± 0.10	7.58 ± 0.09	7.62 ± 0.02	7.69 ± 0.08	7.64 ± 0.04	0.975	.471
	w8	7.61 ± 0.09	7.55 ± 0.01	7.54 ± 0.07	7.54 ± 0.11	7.58 ± 0.08	7.62 ± 0.07	0.796	.573
*A*_T‐CF _(mmol/kg_sw_)	w1	3.89 ± 0.26^a^	5.41 ± 0.81^b^	5.99 ± 0.61^b^	3.65 ± 0.32^a^	4.92 ± 0.20^ab^	5.44 ± 0.58^b^	10.361	<.001
	w8	5.54 ± 0.85	5.11 ± 0.47	5.64 ± 0.22	5.02 ± 0.11	4.45 ± 0.05	5.81 ± 0.82	1.272	.338
Buffer capacity (mmol/kg_sw_)	w1	1.25 ± 0.19^bc^	2.58 ± 0.86^ab^	3.04 ± 0.67^a^	0.95 ± 0.36^c^	2.32 ± 0.17^ab^	2.82 ± 0.37^a^	5.051	<.001
	w8	3.59 ± 0.95	3.21 ± 0.60	3.97 ± 0.27	2.98 ± 0.20	2.62 ± 0.02	4.05 ± 0.77	2.357	.104
Test diameter increment (%)	w12	21 ± 6	15 ± 2	17 ± 2	15 ± 3	14 ± 1	10 ± 5	2.675	.075
Sea urchin diameter with spines increment (%)	w12	19 ± 3^a^	11 ± 2^ab^	9 ± 4^b^	16 ± 4^a^	15 ± 3^a^	2 ± 3^b^	8.965	.001
Jaw size increment (mm)	w12	0.69 ± 0.10^a^	0.33 ± 0.06^b^	0.31 ± 0.05^b^	0.38 ± 0.11^b^	0.32 ± 0.06^b^	0.27 ± 0.03^b^	17.064	<.001
								*F_5,11_	

Note

ANOVA results showing *F* statistic and *p*‐values are shown. pH_NIST‐CF_, pH of the coelomic fluid in NIST scale; *A*_T‐CF_, total alkalinity of the coelomic fluid, buffer capacity: (∆*A*_T_ = *A*_T‐CF_ − *A*_T‐SW_). Significant differences between means of treatments are indicated by letters in superscript; means sharing the same superscript are not significantly different (*p*‐Tukey ≥ .05).

Respiration rate and pH_NIST‐CF_ did not vary between treatments at any time (*p* > .13) and ranged between 0.72 and 1.28 µmol O_2_ hr^−1 ^g^−1^ or between 7.54 and 7.69 pH units, respectively (Table 2). Buffer capacity of the CF (i.e., Δ*A*_T‐CF_ = *A*_T‐CF_–*A*_T‐seawater_) was significantly higher at pH_T_7.4 at w1 (*p* < .001) but it was not affected by treatment at w8 (Table 2).

### 3.2 Microhabitat conditions

Coastal pH_T‐SW_ was 8.10 ± 0.05 (*n* = 4) while tide pool pH_T‐SW_ ranged from 7.4 to 8.8 and varied with time and compartment (S5 in Appendix S1). pH_T‐SW_ below the sea urchins was significantly lower than that over them during the day low tides (*p* ≤ .013), but not during the night low tides (S5 in Appendix S1, Figure S3). Seawater *A*_T‐SW_ was not affected by time nor by compartment (S5 in Appendix S1).

### 3.3 No‐flow biomechanics

#### 3.3.1 Individual and tube foot disk adhesive properties

None of the disk properties varied with treatment at any time (Table S4). Sea urchin detachment force significantly differed with treatment only at w1 (*p* = .013), but not compared to the control. Interestingly, whereas *F*_urchin_ declined with time, *T*_disk_ remained stable.

#### 3.3.2 Tube foot stem mechanical properties

**Figure 2:**
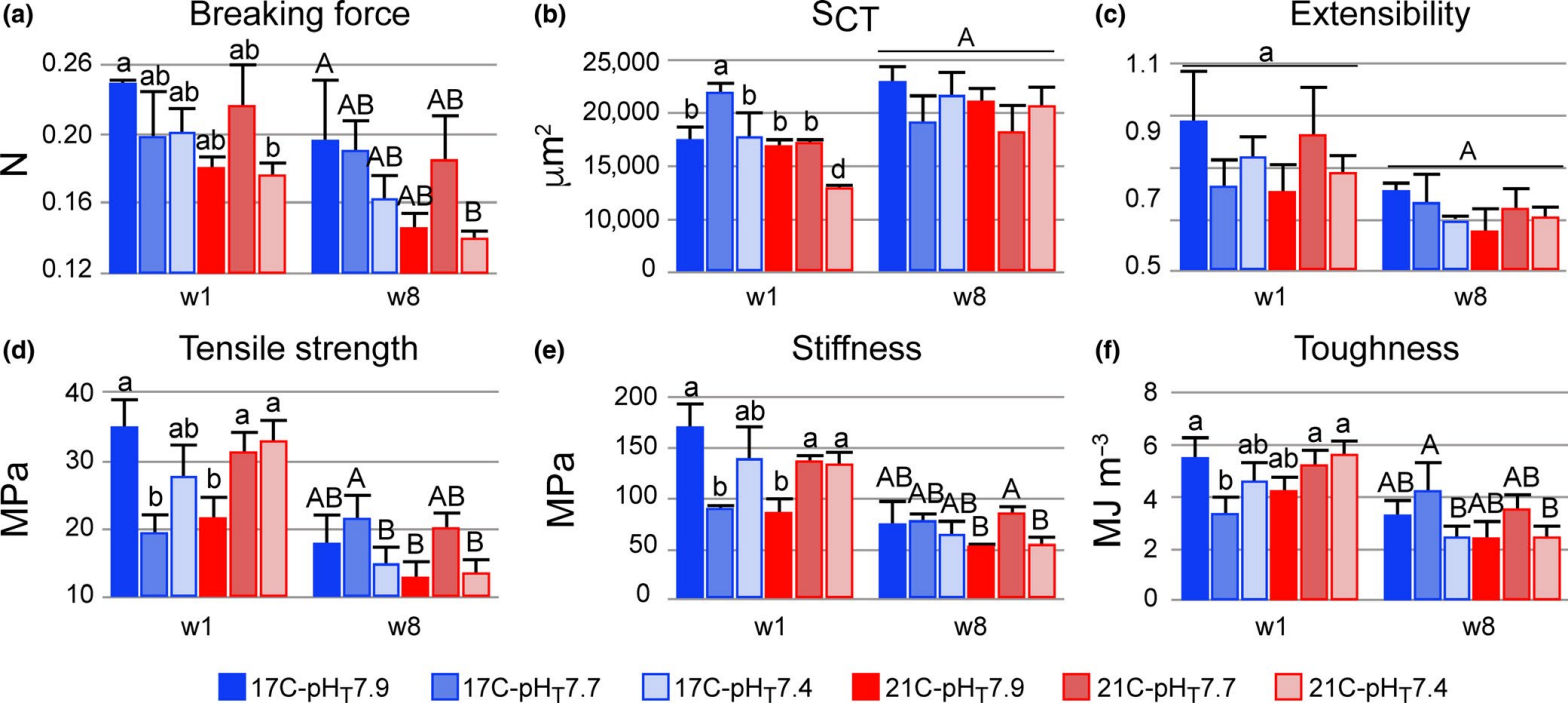
Tube foot mechanical properties (mean ± *SD*, *n* = 3) of *Paracentrotus lividus* per treatment at weeks 1 (w1) and 8 (w8). (a) Breaking force (N), (b) *S*_CT_: cross‐sectional surface area of the stem connective tissue layer (µm^2^), (c) extensibility (unitless), (d) tensile strength (MPa), (e) stiffness (MPa), and (f) toughness (MJ/m^3^). Significant differences between treatments for w1 or w8 are indicated by lowercase or uppercase letters, respectively; means sharing the same letter are not significantly different (*p*‐Tukey ≥ .05)

Breaking force varied significantly at w1 (*p* = .018) and at w8 (*p* = .020), but only between the two extreme treatments (17°C‐pH_T_7.9 vs. 21°C‐pH_T_7.4, Figure 2a). *S*_CT_ differed significantly only at w1 (*p* < .001), being the largest at 17°C‐pH_T_7.7 and the smallest at 21°C‐pH_T_7.4 compared to the control (Figure 2b). Extensibility did not differ between treatments at any time (Figure 2c). Tensile strength, stiffness, and toughness (Figure 2d–f) showed significant differences at both times (*p* < .021; Table S5). At w1, these properties were the highest in the control and differed significantly from those at 17°C‐pH_T_7.7, in which the lowest values were observed. At w8, these properties were not significantly different between the control and other treatments.

### 3.4 In‐flow biomechanics

**Figure 3:**
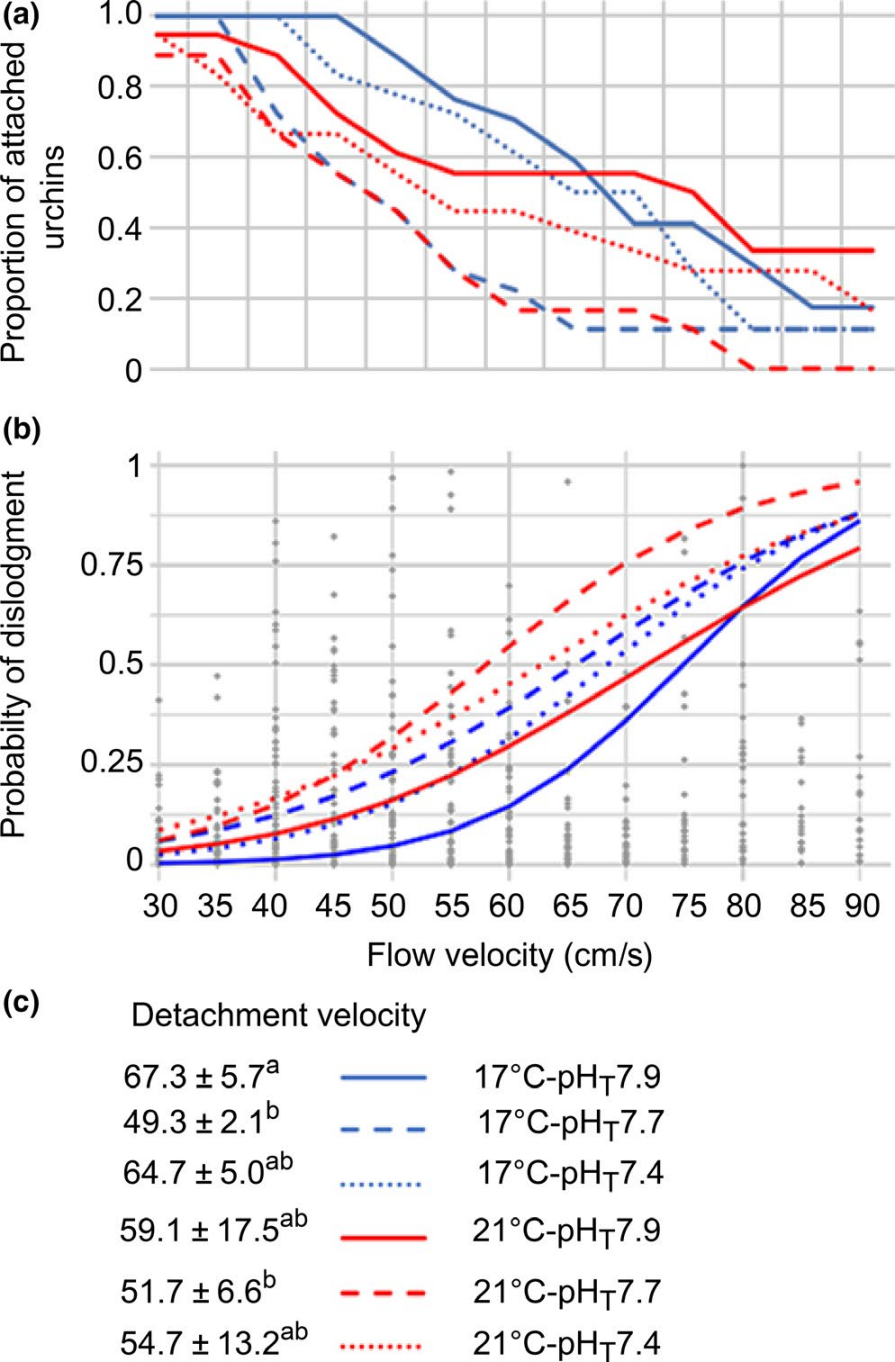
(a) Proportion of attached *Paracentrotus lividus* per flow velocity (cm/s) and treatment. (b) Probability of dislodgement, gray dots represent probability of detachment per flow, while lines reflect their mean values per treatment. (c) Detachment velocity according to treatment (*n* = 3), means sharing the same superscript are not significantly different (*p*‐Tukey ≥ .05)

In the flume tank, individuals detachment started to occur at a flow velocity of 40 cm/s (*V*_F_40) at 17°C, while at 21°C detachment started at *V*_F_30 (Figure 3a). At pH_T_7.7, 40% of sea urchins detached at slow flows (*V*_F_45). Mean detachment velocity (*V*_Det_; Figure 3c) significantly varied with treatments (*F*_5,85_ = 3.54, *p* = .006). Control animals detached at the fastest flow (*V*_Det_ = 67.3 ± 5.7 cm/s), while those at pH_T_7.7 detached at the slowest flow for both 17°C (*V*_Det_ = 49.3 ± 2.1 cm/s, *p*‐Tukey = .017) and 21°C (*V*_Det_ = 51.7 ± 6.6 cm/s, *p*‐Tukey = .046). *V*_Det_ in every treatment was modulated by the density of attached TF (*p* = .007) and by the interaction of both shape variables (spine° circularity: .003 ≤ *p *≤ .007, estimate = 1.13; circularity is a shape index, see S1 in Appendix S1) and movement variables (*V*_Mov_‐Dir_Mov_: .044 ≤ *p* ≤ .045, estimate = −0.005; Table S2, Figure S4). The probability of dislodgment increased with *V*_F_ for every treatment (Figure 2b, Table S1), being the lowest in control conditions while the highest at 21°C‐pH_T_7.7 (Figure 3b); this probability was higher for wider and taller individuals presenting longer spines (Table S1).

### 3.5 Active movement

Active movement velocity (*V*_Mov_) was affected by the interaction between treatment and *V*_F_ (*p* < .047; Table S3, Figures S5 and S6). Sea urchins reduced their *V*_Mov_ with increasing *V*_F_ in all treatments. The combined effect of temperature and pH increased *V*_Mov_ at 17°C‐pH_T_7.7 (*p* < .001), 21°C‐pH_T_7.9 (*p* < .001), and 21°C‐pH_T_7.7 (*p* = .017), while it did not affect *V*_Mov_ at pH_T_7.4 (*p* > .051; Table S3, Figure S7). The median maximal *V*_Mov_ was higher with increased temperature for pH_T_7.9 (17°C:0.059 cm/s; 21°C:0.123 cm/s), pH_T_7.7 (17°C:0.071 cm/s; 21°C:0.100 cm/s), and pH_T_7.4 (17°C:0.091 cm/s; 21°C:0.101 cm/s). According to confidence intervals, individuals stopped moving at *V*_F_65 in the control and at 17°C‐pH_T_7.4 while in the other treatments they stopped at V_F_55.

**Figure 4:**
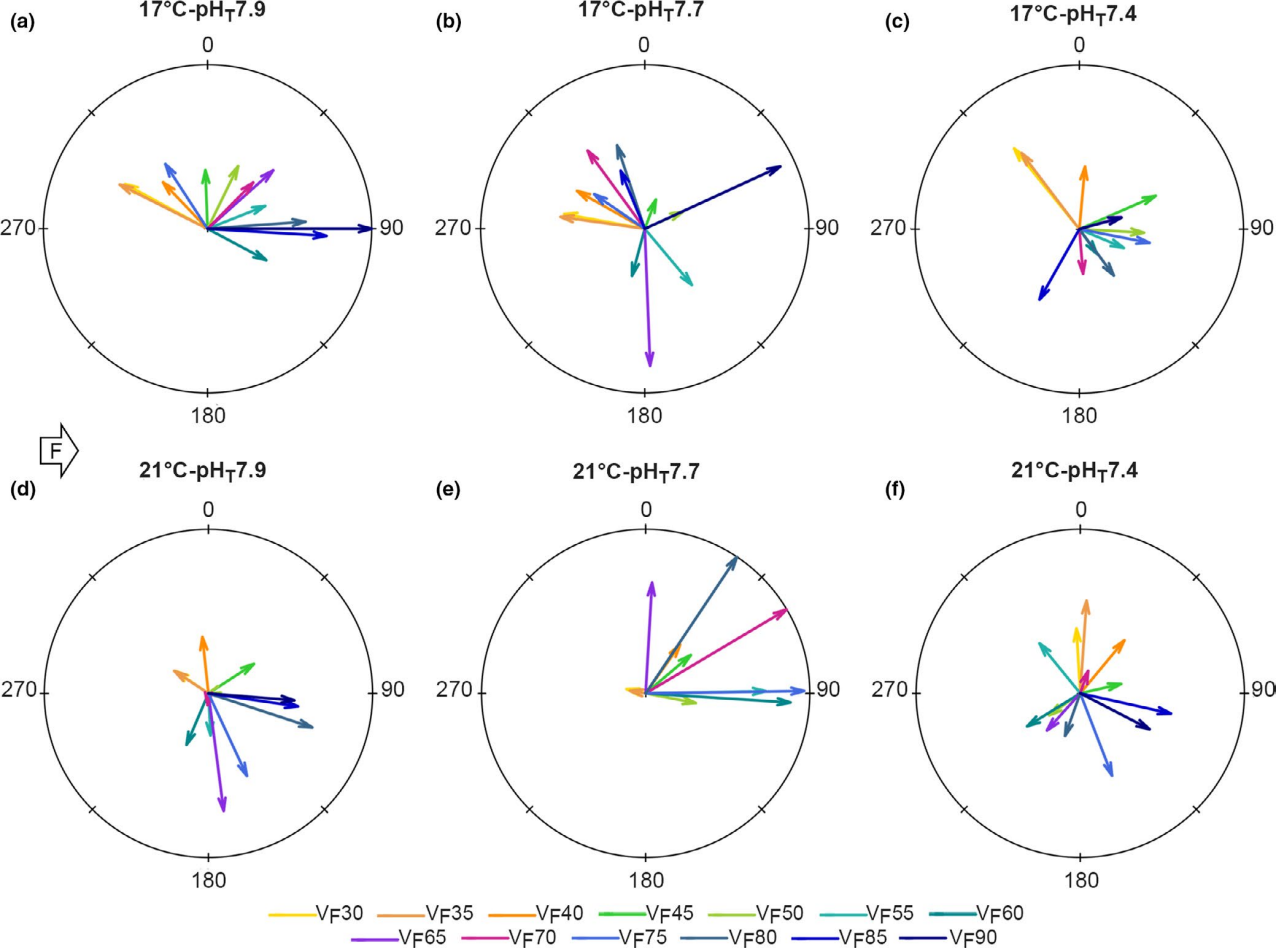
Mean vectors of displacement direction (in degrees) per flow velocity (V_F_) and treatment in *Paracentrotus lividus*. Colored arrow length is inversely proportional to data dispersion. White arrow showing the flow provenance (F), with angles between 0 and 180° implying a downstream displacement direction and angles between 180 and 360 an upstream displacement. Displacement direction at 17°C‐pH_T_7.9 from Cohen‐Rengifo et al. (2018)

At initial flow velocities (*V*_F_30–*V*_F_35), the proportion of sea urchins moving upstream was >78% in the 17°C treatments, but <59% in the 21°C treatments (Figure S8). At pH_T_7.9 (Figure 4a,d), individuals shifted to the downstream at *V*_F_50 at 17°C (*p*‐Tukey_Moore's_Test_ = .02) and at *V*_F_45 at 21°C (*p*‐Tukey_Moore's_Test_ = 0.002). At pH_T_7.7, the relationship between *V*_F_ and direction of movement (Dir_Mov_) is poor and nonsignificant. Indeed, at 17°C (Figure 4b) there is no clear shift in direction, while at 21°C most sea urchins moved downstream (Figures 4e and S8). At pH_T_7.4, a significant shift in movement direction occurred sooner than in the control, at *V*_F_40 (at 17°C, *p*‐Tukey_Moore's_Test_ = 0.002 and at 21°C, *p*‐Tukey_Moore's_Test_ = 0.02). But, from *V*_F_40 on, the small lengths of vector (Figure 4c,f) indicate that individuals got progressively dispersed; hence, Dir_Mov_ seemed disconnected from *V*_F_.

### 3.6 Attached tube feet

**Figure 5:**
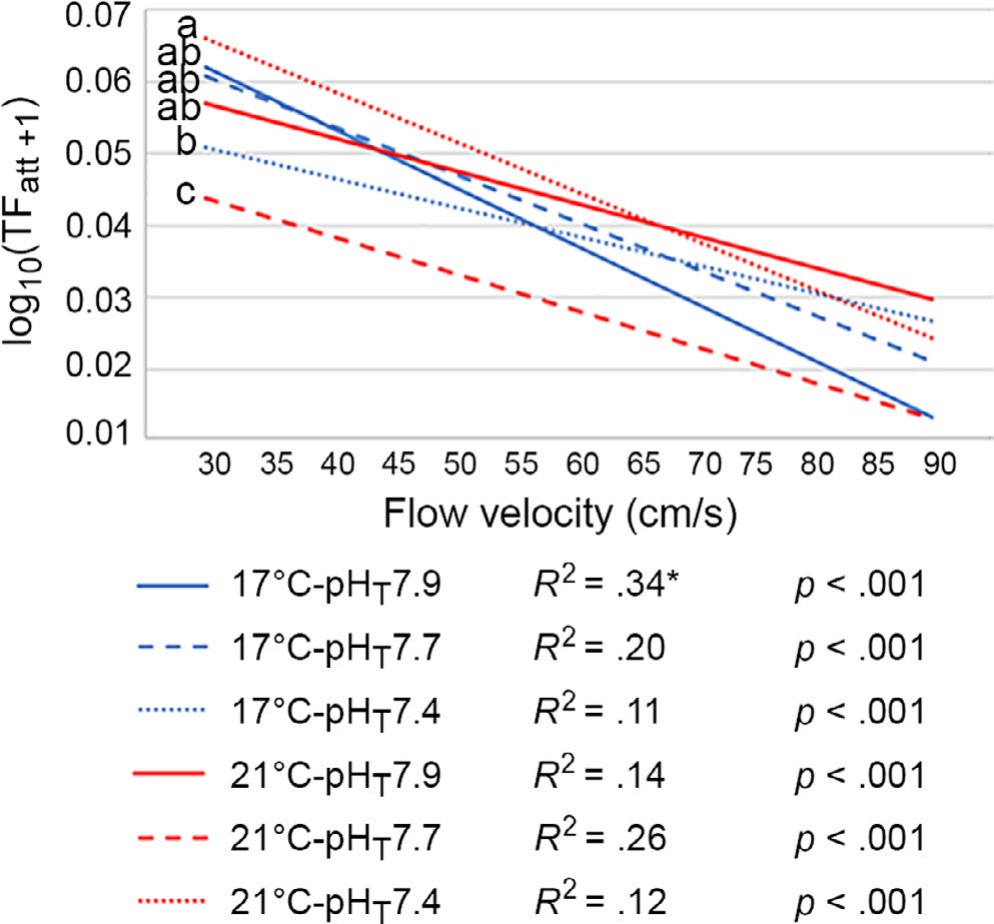
Regression slopes with *R* ^2^ and *p*‐values for the density of total attached TF relative to ambital test surface (TF_att_) per treatment and flow velocity. Data were transformed with $X^{\prime }=\log_{10}\left(X+1\right)$. Significant differences between treatments are indicated by letters; means sharing the same letter are not significantly different (*p*‐Tukey ≥ .05). *Data from Cohen‐Rengifo et al. (2018)

The density of total attached TF (TF_att_) decreased with *V*_F_ (*p*_reg_ ≤ 0.001) in all treatments (Figure 5), though V_F_ only accounted for 11%–34% of the variation. TF_att_ significantly differed between treatments (*F*_5,754_ = 10.8, *p* < .001), being the lowest at 21°C‐pH_T_7.7. The percentage of detached TF at *V*_F_90 was the highest (83%) in the control and the lowest (42%) at 17°C‐pH_T_7.4 (Table S6).

### 3.7 Shape and spine analyses

**Figure 6:**
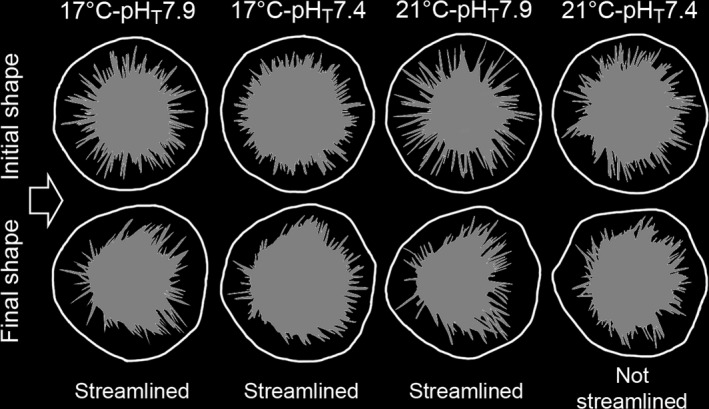
Initial and final shape of *Paracentrotus lividus* for treatments in which shape varied significantly with flow velocity. Reconstructed outlines in white and planform shape in gray. Arrow represents flow provenance

The shape outline of the sea urchins differed according to *V*_F_ and treatments (*F*_two‐way_MANOVA(30,210)_ = 1.33, *p* ≤ .001). Variation in shape according to V_F_ was not significant at pH_T_7.7 for both temperatures (*p*_pairwise_MANOVA_ ≥ .37). In the control, shape significantly started to change at *V*_F_60 (*p*_pairwise_MANOVA_ ≤ .001) and was mainly modulated by homogeneous movements of upstream spines toward the downstream, that is, symmetrically to the right and left of the flow (Figure 6). At 17°C‐pH_T_7.4, shape started to change at *V*_F_55 (*p*_pairwise_MANOVA_ = .016), but the final streamlined shape was poor (Figure 6). At 21°C‐pH_T_7.9, shape variation occurred at *V*_F_50 (*p*_pairwise_MANOVA_ = .011), and the streamlined shape was more pronounced than in the control (Figure 6). Finally, at 21°C‐pH_T_7.4, shape started to change at *V*_F_50 (*p*_pairwise_MANOVA_ = .024), but as spines situated upstream moved asymmetrically (Figure 6) and spines situated perpendicular to the flow moved very little (*p*_Moore's_Test_ > 0.2), the resulting shape was not streamlined.

Spine angle (Spine°) analysis revealed that spines situated perpendicular to the flow moved significantly downstream at *V*_F_60 in the control (*p*_Moore's_Test‐VF30_Vs_VF60_ = .04) and at *V*_F_65 (*p*_Moore's_test‐VF30_Vs_VF65_ = .03) at 17°C‐pH_T_7.4. At 21°C‐pH_T_7.9, spine° differed between *V*_F_35–*V*_F_70 (*p*_Moore's_Test_ = 0.03) and *V*_F_50–*V*_F_60 (*p*_Moore's_Test_ = .03) indicating an unclear shift in spine orientation. Spine° did not vary with *V*_F_ at pH_T_7.7 for both temperatures (17°C:*p*_Moore's_Test_ > 0.6, 21°C:p_Moore's_Test_ > 0.1). Mean Spine° is shown in Figure S10.

## 4 DISCUSSION

The integrative approach of this study revealed that resistance to hydrodynamism in *Paracentrotus lividus* resulted from a complex array of behavioral and mechanical strategies performed to trade‐off for negative effects in physiology, TF biomechanics, and behavior that occurred under a mid‐term exposure to simultaneous OW and OA. Furthermore, our experiments revealed plastic responses that were highly variable according to treatments and increasing flows.

### 4.1 Physiological state and microhabitat preadaptation

Within pits, *P. lividus* experiences dual pH_T‐SW_ values at the same temporal scale, being significantly lower (−0.2 units) below the individual than over it. This difference in pH_T‐SW_ is probably linked to (a) the absence of water motion at low tide which restrains water renewal and gas exchange and to (b) the respiratory activity of the rock biofilm and the sea urchin. So, the natural pH and temperature diel fluctuations (Kwiatkowski et al., 2016; Moulin, Catarino, Claessens, & Dubois, 2011; Truchot & Duhamel‐Jouve, 1980), together with the dual pH conditions, can explain *P. lividus* plastic physiological responses observed under a broad pH spectrum, and the tolerance to experimental chronic low pH that allows to maintain stable both coelomic fluid pH and respiration rate (Catarino et al., 2012; Collard et al., 2013; Collard, Dery, Dehairs, & Dubois, 2014).

Jaw and sea urchin (test diameter with spines) growths were 2.6‐fold and 2.5‐fold, respectively, lower in the treatments compared to the control. Coping with warming and extracellular acidosis over the course of 8 weeks could have led to a lower resource allocation to growth (Hofmann & Todgham, 2010; Stumpp, Trübenbach, Brennecke, Hu, & Melzner, 2012). Although individuals were fed equally in every treatment, a possible modified digestive efficiency due to OW and/or OA (not measured) could account for the observed growth differences.

### 4.2 No‐flow tube foot mechanical performance

At w1, control breaking force is comparable with values previously reported for *P. lividus* (Cohen‐Rengifo et al., 2017). Overall, mechanical properties showed no clear pattern at w1, which could reflect the different individual rates of acclimation to seawater changes. At w8, stems at 17°C‐pH_T_7.7 can absorb more energy during deformation (toughest stems) and therefore are more resistant to an external load (strongest stems). In contrast, at 21°C‐pH_T_7.4 (extreme) stem breaking force seemed deteriorated, being 1.5‐fold lower than that in the control. In the starfish *Asterias rubens*, TF strength was not affected by pH_T_7.4, while several mechanical properties of the byssus threads in the bivalves *Pinctada fucata* and *Mytilus trossulus* (O'Donnell et al., 2013) were reduced probably due to a shift in energy allocation or to a pH‐induced alteration during the adhesive curing process (Collard et al., 2013; George & Carrington, 2018; Li et al., 2017; O'Donnell et al., 2013).

The lower adhesive strength of whole individuals observed at w8 compared to w1 could be related, to some extent, to a downregulation of the adhesive protein Nectin under experimental conditions (Toubarro et al., 2016). However, since neither disk tenacity nor disk detachment force declined with time, reduced adhesive strength of the whole individual is more likely due to a lower number of attached TF, in response to a lack of external stimuli (Cohen‐Rengifo et al., 2018) under no‐flow conditions.

It is important to highlight that a sevenfold higher force is needed to break the stem than to detach the disk from the substratum, meaning that, under a given external load, tube foot detachment can occur before tube foot breakage. It is probably more cost‐effective to produce new adhesive compound than to lose an entire adhesive organ. Support for this statement comes additionally from nonquantified observations during flume tank trials in which TF detachment involved very low rates of stem breakage. In addition, the fact that mechanical properties of the stem decrease with time, while adhesive properties of the disk did not, can indicate that mechanical properties of the stem played a less relevant role in attachment capacity than adhesive properties of the disk. Indeed, the noncovalent adhesive and cohesive interactions between the adhesive compound and the substratum and, within the adhesive itself, could be influenced by pH, but larger pH changes would be necessary to significantly modify these interactions (Flammang, Demeuldre, Hennebert, & Santos, 2016). Our observation that adhesion strength is not influenced by reduced pH seems to corroborate this hypothesis. In the light of this, sea urchin dislodgement should be mainly modulated by behavioral responses to cope with the impact of high temperature and low pH.

### 4.3 In‐flow behavioral strategies in warmer and more acidic ocean

Three main behavioral strategies were observed to avoid detachment: (a) improving TF attachment, implemented during the whole flow velocity range; (b) escaping the flow, at slow flow velocities (*V*_F_30–*V*_F_45); and (c) streamlining, at fast flow velocities (*V*_F_50–*V*_F_90). The latter two have been already reported for *P. lividus* under current seawater conditions by Cohen‐Rengifo et al. (2018).

#### 4.3.1 Improving tube foot attachment strategy

Intuitively, the first reaction of an organism that depends on its adhesive appendages to resist flow is by using them. According to our results, a first behavioral strategy, when climate change had mechanically weakened these organs, was to compensate by increasing the number of attached TF or by controlling the percentage of detached TF. This either amplifies the total adhesive force or avoids its decrease. The mean percentage of detached TF at V_F_90 was lower in every climate change treatment, being even halved at 17°C‐pH_T_7.4 (−42%) with respect to the control (−83%). In the extreme condition, animals initially improved the density of attached TF, but the persistent hydrodynamic stress combined with OW and OA was detrimental (TF detachment up to 69%) and lead to earlier detachment.

#### 4.3.2 Escaping strategy

*V*_Mov_ increased with temperature at pH_T_7.9 and pH_T_7.7. Enhanced locomotion and activity have been observed in response to warming (Kidawa, Potocka, & Janecki, 2010; Pewsey, 2004; Young, Peck, & Matheson, 2006) and acidification (Cripps et al., 2011; Manríquez et al., 2013; Nilsson et al., 2012; Spady, Watson, Chase, & Munday, 2014), but there is a poor understanding about the effects of combined stressors. As with the echinoid *Loxechinus albus* (Manríquez et al., 2017), our results revealed positive synergistic effects of OW and OA, since sea urchins were driven to adopt a riskier behavior by moving faster despite the hydrodynamic stress. On the contrary, negative synergistic effects of OW and OA were reported on locomotion of jumbo squid (Rosa & Seibel, 2008) and a decapod (Dissanayake & Ishimatsu, 2011) as a result of reduced metabolic scope.

At slow flow velocities, sea urchins reared under climate change conditions displayed an escaping behavior by moving fast, looking for less hydrodynamically stressful zones. Yet, their movement stopped sooner, probably because they cannot move safely anymore. In the control, at *V*_F_50, 31% of sea urchins moved at the slowest *V*_Mov_ and stopped moving at *V*_F_65 (Cohen‐Rengifo et al., 2018). This proportion increased to 50%–69% in individuals reared under climate change conditions, in which a complete interruption of movement occurred mainly at V_F_55.

Modified behavior resulted from three main pathways: elevated metabolic load, avoidance behavior away from the localized source of change, and information disruption (Briffa, Haye, & Munday, 2012; Lürling & Scheffer, 2007). The observed negative effects on growth and on behavior were probably due to an overall increase in metabolic load to cope acidosis and a shift in energy allocation. In addition, the implementation of an escaping behavior suggests that detection of the hydrodynamic stressor was well accomplished but as sea urchins adopted a riskier behavior, their interpretation of the hazardous hydrodynamic conditions may have been altered. Indeed, in marine vertebrates and crustaceans, behavioral changes often take place through info‐disruption that occurs when their ability to gather and assess information and consequently their decision‐making are impaired (Briffa et al., 2012; de la Haye, Spicer, Widdicombe, & Briffa, 2011).

Control *P. lividus* favored a mean downstream displacement at fast flows while in the other treatments, movement was random and characterized by constant back and forth. A change in movement patterns in acidified waters has been observed in a teleost probably because CO_2_ affected its neurophysiology or because CO_2_ was detected as a constant stressor (Green & Jutfelt, 2014).

#### 4.3.3 Streamlining strategy

Whereas spine movements led to a change in the overall shape, the direction and amplitude of these movements determined whether the final shape was streamlined or not. For instance, at 21°C‐pH_T_7.4 even though shape significantly changed, the final shape was not streamlined (Figure 6). The lack in coordination between spines movements and shape modification demonstrated that individuals displayed streamlining behavior in an atypical way. Although atypical or absent streamlining is detrimental for detachment, it can also favor feeding behavior as spines in a “up position” can capture drifting algae.

Control *P. lividus* displayed a first reaction to increasing flows at *V*_F_35 as observed in *Strongylocentrotus franciscanus* that perceives flow variations at flow velocities as slow as <10 cm/s, a behavior that hitches always leads to a streamlining behavior (Stewart & Britton‐Simmons, 2011). In the climate change treatments, no early reaction to flow was observed in *P. lividus*, attesting for a possible disruption in information processing. In various organisms, behavioral abnormalities have been attributed to changes in seawater chemistry that leads malfunctions in neurological mechanisms (such as information processing) involving type A γ‐aminobutyric acid (GABA_A_) receptors (Chivers et al., 2014; Domenici et al., 2012; de la Haye et al., 2011; Nilsson et al., 2012; Tuomainen & Candolin, 2011). These ionotropic receptors present a high conductivity for Cl^−^ and for ${\text{HCO}}_{3}^{-}$ (Bormann, Hamill, & Sakmann, 1987; Nilsson et al., 2012). Echinoids including *P. lividus* protect themselves against acidosis through accumulation of ${\text{HCO}}_{3}^{-}$ in the extracellular fluid, inducing compensatory reductions in Cl^−^ (Collard et al., 2014; Miles, Widdicombe, Spicer, & Hall‐Spencer, 2007; Stumpp et al., 2012). The excitatory action of GABA resulting in increased Cl^−^ has been already reported in echinoid tube feet (Florey, Cahill, & Rathmayer, 1975). We hypothesize that high CO_2_ could alter information processing through the GABA_A_ pathway, leading to the behavioral modifications observed in *P. lividus*.

#### 4.3.4 Integration of responses and ecological implications

Responses facing simultaneous OW and OA did not follow an intuitive pathway and were sometimes conflicting, making difficult to identify causalities and to discriminate the drivers governing one behavior or another. For instance, animals held at 21°C‐pH7.7 showed improved TF mechanical properties but worst behavioral performance than animal held at 21°C‐pH7.4. This might indicate that the biochemical paths and neurological mechanisms operate better in acidic environments. Likewise, sea urchins that attached with the lowest density of TF (21°C‐pH_T_7.7) did not move consequently at the lowest velocity, but on the contrary, showed the same median maximal velocity as individuals that attached with the highest number of TF (21°C‐pH_T_7.4). Therefore, TF_att_ did not modulate the velocity at which sea urchins moved, at least not directly or not only by itself.

**Figure 7:**
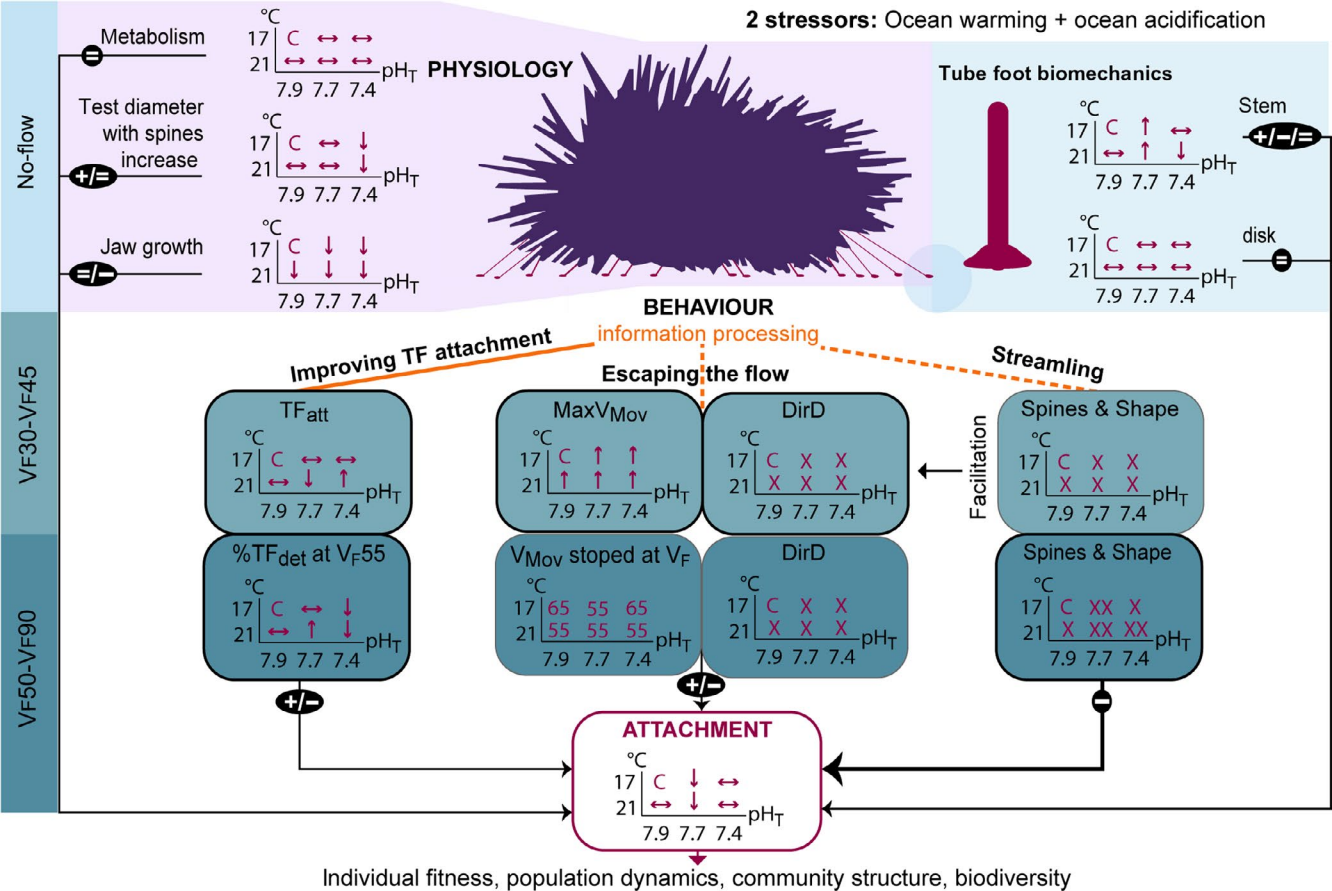
Conceptual framework showing physiological, mechanical, and behavioral responses favoring (+), impairing (−), or not affecting (=) sea urchin attachment under ocean warming (OW) and acidification (OA). Sea urchins were subjected to 6 treatments including two temperatures (17°C and 21°C) and three pH_T_ (7.9, 7.7, and 7.4). Compared to the control (C), responses increased (↑), decreased (↓), did not change (↔), were atypical (X), or not achieved (XX). Dashed lines stand for deficiency. Physiology and tube foot biomechanics were measured under no‐flow, while behavior was evaluated under increasing flow velocity (*V*_F_: 30–90 cm/s). To avoid dislodgement, three behavioral strategies were undertaken: improving tube foot attachment, escaping the flow, and streamlining. See list of abbreviations in Appendix S2

Figure 7 provides a conceptual framework showing the effects of OW and OA on physiology, biomechanics and behavior according to flow velocity. Weakened TF stems and unaltered TF disks under OW and OA seemed to have a moderate role in sea urchin overall attachment. Concerning physiology, the energy flux to maintain acid‐base balance probably disfavored energy allocation for skeleton and spine growth. However, under increased hydrodynamics, a smaller size can reduce dislodgement risk. Alteration in information processing leads to varied behavioral modifications. To avoid detachment in a warmer and more acidic environment with simultaneous increase in flow velocity, sea urchins implemented a first strategy by increasing the density of attached TF and controlling the percentage of TF detachment. Info‐disruption could have altered risk assessment of the hydrodynamic stress and decision‐making, and drove animals through two routes. First, at slow flow velocities (*V*_F_30–*V*_F_45), spines did not react normally to flow variations, favoring the second strategy, escaping the flow. Echinoids in every climate change treatment moved faster to optimize shelter search. Yet, their movement stopped sooner. Second, at fast flow velocities (*V*_F_50–*V*_F_90), the third strategy, streamlining was performed atypically or was not achieved at all. In both cases, this accounted for an earlier dislodgement.

When animals experience an environmental change, their earliest response is often a plastic modification of their behavior (Tuomainen & Candolin, 2011). The success of the behavioral change will depend on the rapidity of their reaction (Price, Qvarnstro, & Irwin, 2003) and whether populations have or not encountered similar conditions during their recent evolutionary history (Ghalambor, McKAY, Carroll, & Reznick, 2007). Adjustments in behavior could be beneficial if they improve fitness by increasing survival or reproductive success (Tuomainen & Candolin, 2011). Moving fast to escape the flow can be considered as an adaptive response as it reduces the probability of dislodgment and therefore improves survival. On the contrary, atypical or impaired streamlining can be maladaptive as it causes earlier dislodgment which reduces survival. Individuals showing plasticity are often selected to constitute populations that can survive rapid environmental changes and ensure population persistence (Kinnison & Hairston, 2007), which might be the case for the *P. lividus* Aber population.

In intertidal pits, sea urchins experience fluctuating pH that is often close to 7.7 or even lower below the animal. Whereas this reality induced adaptive physiological and mechanical responses when only two stressors were evaluated (OW and OA), maladaptive behavioral responses appeared when another factor (hydrodynamics) was included. In the near‐future, *P. lividus* inhabiting pits will experience more severe pH/pCO_2_ conditions. However, this could be partially compensate by diel natural fluctuations since animals will be exposed progressively to reduce pH (Jarrold, Humphrey, McCormick, & Munday, 2017). It is probable that *P. lividus* would successfully face pH stress, yet the outcome of the interaction with a fluctuating temperature remains unknown.

## CONFLICT OF INTEREST

None declared.

## AUTHORS CONTRIBUTION

MC‐R, PF, and PD conceived the project. MC‐R, AA, TB, PF, and PD provided ideas and initiatives. MC‐R, AA, TB, PF, and PD designed the experimental design and setup. MC‐R, AA, and SM collected and treated the data. MC‐R, AA, and PD conceived the statistical treatments. MC‐R led the writing of the manuscript. All authors contributed significantly to the drafts and gave final approval for publication.

## Data Availability

Datasets are available in https://doi.org/10.5061/dryad.123t3gr.
