# Supplementary material for: Ocean warming and acidification alter the behavioral response to flow of the sea urchin Paracentrotus lividus
Source: Ecol Evol. 2019 Oct 17;9(21):12128–43. doi: 10.1002/ece3.5678 (PMC6854335; doi:10.1002/ece3.5678)
Supplement: Supplementary file 1 [file ECE3-9-12128-s001.docx]

**SUPPLEMENTARY INFORMATION**

**Ocean warming and acidification alter the behavioural response to flow in the sea urchin *Paracentrotus lividus***

Mishal Cohen-Rengifo^1,2*^, Antonio Agüera^2♦^, Tjeerd Bouma^3^, Saloua M’Soudi^2^, Patrick Flammang^1^ and Philippe Dubois^2^

^1^Université de Mons, Institut des Biosciences, Laboratoire de Biologie des Organismes marins et biomimétisme. 23 Place du Parc, 7000 Mons, Belgium

^2^Université Libre de Bruxelles, Laboratoire de Biologie Marine (CP160/15). 50 Av. F.D. Roosevelt, 1050, Brussels, Belgium

^3^ Royal Netherlands Institute for Sea Research (NIOZ), Department of Estuarine and Delta systems, and Utrecht University, P.O. Box 140, 4400 AC Yerseke, The Netherlands

^♦^Current affiliation: Institute of Marine Research, Austevoll Research Station, 5392 Storebø, Norway

**S1. Methodological details concerning shape analysis and shape indices calculation**

Planform pictures used for shape analysis were binarized so that, the sea urchin surface area is white while the background is black. Pictures were analysed in Matlab v2015 software using Image Processing ToolboxTM. The following size descriptors were measured according to Agüera and Brophy (2011) and Cohen-Rengifo *et al.* (2018): Area (A, mm^2^) the total number of white pixels in the binary image; perimeter (P, mm^2^), the number of pixels in a 1 pixel-wide outline enclosing the white area; and sea urchin length in the X (LX, mm) and Y (LY, mm) axis (corresponding to test diameter and height with spines for profile surfaces, and to length parallel and perpendicular to flow for planform surfaces). Based on these size descriptors, the following shape indices were calculated:

Aspect Ratio = LX⁄LY

Circularity = A⁄P^2^

Ellipticity = (LX-LY)⁄(LX+LY)

Form Factor = 4πA⁄P^2^

Rectangularity = A⁄(LX*LY)

Roundness = 4A⁄(πLX^2^ )

Scale was not kept constant among the individuals, which impedes direct analysis of size descriptors. However, this had no effect on shape indices as they are dimensionless ratios. Therefore, only shape indices were considered for further statistical analyses (S4).

**S2. Methodological details concerning physiological measurements**

At week 1 and 8, pH_NIST-CF_, A_T-CF_ (mmol kg_SW_^-1^) and respiration rate (μmol h^-1^ g_SW_^-1^) were assessed in 3 individuals per aquarium. 0.5 ml of CF were extracted with a Myjector 0.5 ml insulin syringe by puncture through the peristomial membrane. pH_CF_ was immediately measured with a 827 pH Metrohm meter mobile with a combined Metrohm LL biotrode (Metrohm, AG, Switzerland) both previously calibrated with CetriPUR® buffers 4 and 7 (Merck KGaA, Germany). A_T-CF_ was measured according to Gran's (1950) potentiometric titration modified for micro-measurements (Collard *et al.*, 2013) and standardised with Dickson laboratory (University of California) reference material (Batch 135, measures were within ± 6% of the reference value). Respirometry assays were conducted and corrected according to Collard et al., (2013).

At week 1 and 12, sea urchin diameter (d_urchin_, with spines), test diameter (d_test_) and height (h_test_) were measured with a standard caliper (precision 0.1 mm) in 6 alive individuals per aquarium. Ambital spine length (l_spine_) was calculated as half the difference between d_urchin_ and d_test_. The cleaned demy-pyramid length was measured from the oral tip to the epiphysis junction (Ebert, 1980; Ebert *et al.*, 2014) with a standard caliper (precision 0.1 mm). Somatic growth for d_urchin_, d_test_, and h_test_ was calculated using mean values per aquarium as their difference between week13-week1 divided by week1 (week13-week1/week1). To assess skeletal growth, calcein-tagged demi-pyramids were observed under UV light using a Leica DM IRB inverted research microscope and a micrometric ocular set at 5X (Leica Microsystems IR GmbH, Germany). Jaw size increment (mm) between the calcein tag and the distal epiphysis junction of the demi-pyramid was measured (Edwards & Ebert, 1991).

**
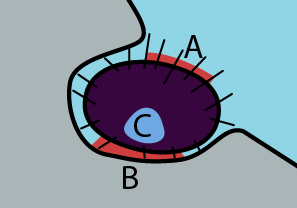
Figure S1**. Simplified illustration of a sea urchin self-burrowed pit showing the compartments were samples of liquid were collected for pH_T_ and A_T_ evaluation. Seawater samples were collected from the sea urchin surface, between spines just at the entrance of the pit (**A)** and below the sea urchin, close to pit internal wall (**B**), while coelomic fluid samples were extracted from the sea urchin coelomic cavity (**C**).

**S3. Methodological details concerning microhabitat conditions**

In June 2016, pH_T_ and A_T_ were measured within pits in intertidal pools. Samples were collected 2 h before and 2 h after the day and the night low tide (LT) from 3 compartments: over, below and inside (i.e. the coelomic fluid) the urchin (Fig S1). Twelve random pits hosting sea urchins before the LT and another 12 after the LT were selected to extract samples from over and below, while CF samples were extracted only from half of these individuals. Samples of pool seawater were taken once before and once after the day and night LTs. Two intertidal pools were selected based on a previous characterization made by Claessens (2009) and Moulin et al., (2011). The seawater pH of the big pool (B-pool, 5 x 1.5 x 0.5 m) fluctuate little over a diel cycle, while that of the small pool (S-pool: 2.4 x 0.6 x 0.3 m) fluctuate more over a diel cycle. B-pool was sampled at every time, while S-pool only 2 h after night LT. Coastal pH was measured once a day.

Sea urchin coelomic fluid (~0.5 ml) was collected with a Myjector 0.5 ml insulin syringe by puncture through the peristomial membrane. Around 0.6 ml of seawater was taken above the sea urchin (between the spines) with a HSW 1 ml fine dosing syringes. For samples below the sea urchin a longer needle (21Gx2”-0.x50 mm) was employed. 0.5 ml of each sample were transferred to a 0.5 ml Eppendorf tube. Immediately after collection, pH_T_ of every sample and A_T-CF_ were measured. Seawater samples were fixed with HgCl_2_ 7% (w/vol) to measure alkalinity *a posteriori*. As alkalinity varied ± 8% with respect to the reference value (Dickson laboratory reference material batch 135 and 151, University of California), a correction was applied.

**S4. Methodological details concerning statistical analyses**

Each of the 6 treatments were independently triplicated (1 aquarium = 1 replicate). Within each aquarium, sea urchins were put into 3 different compartments, each of which was designated to measure a particular response variable (physiological, biomechanical or behavioural). ANOVAs, linear regressions and tests for homogeneity of slopes were done using SYSTAT 12 software with α=0.05. When appropriate, ANOVAs were followed by Tukey multiple comparison tests. Generalized linear mixed-effects models were developed with R v3.4.1 software (R Development Core Team 2015) using the packages lme4 and mgcv. Prior to every model, a Pearson correlation matrix was calculated to exclude from the models redundant effect between variables that could affect models’ variance. Shape analyse and circular variables were analysed with R v3.4.1 (R Development Core Team 2015) circular package.

For sea urchin adhesive properties and physiological variables, two-factor nested ANOVAs were carried out with treatment as fixed factor and aquarium as nested random factor. A one-way ANOVA with treatment as fixed factor was carried out for V_Det_ and growth variables, the latter being previously transformed with the function $X^{'}=arcsin\sqrt{X+0.5}$ (Zar, 1999). For mechanical and adhesives properties of tube feet, three-factor nested ANOVAs were carried out with tube foot (random subject) nested within sea urchin (random factor) which are nested within aquarium (random factor, n=3), nested again in levels of treatment (fixed factor). Regarding the microhabitat sampling, pH_T-SW_ and A_T-SW_ (n=12) at B-pool were analysed using two-factor ANOVA model with repeated measures on one cross factor with compartment (fixed repeated factor, 2 levels: below and over) nested within pit (random subject) and crossed with time (fixed, 4 levels: daybeforeLT, dayafterLT, nightbeforeLT and nightafterLT). Comparisons between B-pool and S-pool only at nightafterLT were also performed using the same ANOVA model with pit (random factor) nesting compartment (fixed repeated factor, 2 levels: below and over) crossed with pool (fixed, 2 levels: B-pool, S-pool). The same analysis was carried out to compare pH_T-SW_ and A_T-SW_ with those of the CF (n=6) with the fixed repeated factor compartment including 3 levels (below, over and CF). Treatment effects for ANOVAs were tested against their corresponding between-subjects error term according to Doncaster and Davey (2007).

TF_att_ and TF_att_% were transformed with the function $X^{'}=\log_{10}(X+1)$ and $X^{'}=arcsin\sqrt{X}$, respectively (Zar, 1999). TF_att_ per treatment was analysed by means of linear regressions to determine if TF_att_ and TF_att_% vary with V_F_. Homogeneity of slopes between treatments from the resultant regression lines was done using an ANCOVA with V_F_ as covariate and treatment as numeric categorical factor. ANCOVAs were followed by Tukey multiple comparisons.

Per treatment, Dir_Mov_ was tested for isotropy with an omnibus Kuiper’s test while Spine° with a Reyleight test for unimodal departures from uniformity (Pewsey *et al.*, 2013). Circular mean and standard deviation for Dir_Mov_ and Spine° were calculated per V_F_ for each treatment (Batschelet, 1981; Pewsey *et al.*, 2013). A Moore’s test for paired circular data was performed to determine whether Dir_Mov_ and Spine° vary with V_F_ for each treatment. Because this test is only applicable for 2 samples, the test was carried out for each pair of samples by correcting p-values using Benjamini-Hochberg method (Benjamini & Hochberg, 1995).

Statistical methods for shape analyses followed Jossart (2010) and Agüera and Brophy (2011). A 2-way MANOVA was performed combining principal components of both shape indices and elliptic Fourier coefficients, to seek for a potential effect of V_F_ (fixed factor with seven levels: V_F_30-55 and the maximal V_F_ reached by individuals) and/or treatment (fixed nesting factor with 6 levels). Multiple comparisons were carried out using a pair-wise MANOVA with p-values adjusted using the Benjamini-Hochberg method (Benjamini & Hochberg, 1995).

**Table S1**. Details for Model1 concerning the probability of dislodgement of *Paracentrotus lividus*. Detachment (binomial distribution with 0 meaning no detachment and 1 detachment) was expressed as a function of treatment (treat, fixed factor) and the covariates flow velocity (vf), test diameter (dia), test height (hei), ambital spine length (spine) and flow regime (flow2’ and flow1’). Aquarium (replica) was nested within treatment. P-values codes: ***: p≤0.001, **: 0.001<p≤0.01 *: 0.01<p≤0.05.

|  | **Model 1: Generalized linear mixed-effect model** | | | | |  |
| --- | --- | --- | --- | --- | --- | --- |
| Fit by: | maximum likelihood (Laplace Approximation) | | | | | |
| Family: | binomial ( logit ) | | | | | |
| Formula: | Detachment ~ treat + vf + dia + spine + hei + treat:vf + treat:dia + treat:spine + treat:hei + dia:hei + (1\|aquarium) | | | | | |
| Control: | glmerControl(optimizer="bobyqa") | | | | | |
|  |  | | | | |  |
|  | **AIC** | **BIC** | **logLik** | **deviance** | **df.resid** |  |
|  | 2457.4 | 2641.3 | -1197.7 | 2395.4 | 2751 |  |
|  |  | | | | |  |
|  | **Scaled residuals:** | | | | |  |
|  | Min | 1Q | Median | 3Q | Max |  |
|  | -5.5750 | -0.4623 | -0.1652 | 0.4980 | 5.0458 |  |
|  |  |  |  |  |  |  |
|  | **Random effects:** | | | | |  |
|  | Groups | Name | Variance | Std.Dev. |  |  |
|  | aquarium | (Intercept) | 0.5471 | 0.7397 |  |  |
|  | Number of obs: 2782, groups: aquarium, 18 | | | | |  |
|  |  |  |  |  |  |  |
|  |  |  |  |  |  |  |
| **Fixed effects:** | | | | | | |
| treatment | 17°C-pH_T_7.9 | 17°C-pH_T_7.7 | 17°C-pH_T_7.4 | 21°C-pH_T_7.9 | 21°C-pH_T_7.7 | 21°C-pH_T_7.4 |
| 17°C-pH_T_7.9 |  |  |  | *** | *** | ** |
| 17°C-pH_T_7.7 |  |  |  |  |  | *** |
| 17°C-pH_T_7.4 |  |  |  |  |  | *** |
| 21°C-pH_T_7.9 |  |  |  |  |  | ** |
| 21°C-pH_T_7.7 |  |  |  |  |  | ** |
|  |  |  |  |  |  |  |
|  | 17°C-pH_T_7.9 | 17°C-pH_T_7.7 | 17°C-pH_T_7.4 | 21°C-pH_T_7.9 | 21°C-pH_T_7.7 | 21°C-pH_T_7.4 |
| vf | *** | *** | *** | *** | *** | *** |
| dia | *** | *** | *** | ** | ** |  |
| spine | ** |  | ** |  | *** | * |
| hei | * |  |  | * | *** | *** |
| dia*hei | *** | *** | *** | *** | *** | *** |
|  |  |  |  |  |  |  |
| treat*vf | 17°C-pH_T_7.9 | 17°C-pH_T_7.7 | 17°C-pH_T_7.4 | 21°C-pH_T_7.9 | 21°C-pH_T_7.7 | 21°C-pH_T_7.4 |
| 17°C-pH_T_7.9 |  | * | * | ** |  | *** |
| 17°C-pH_T_7.7 |  |  |  |  |  |  |
| 17°C-pH_T_7.4 |  |  |  |  |  |  |
| 21°C-pH_T_7.9 |  |  |  |  |  |  |
| 21°C-pH_T_7.7 |  |  |  |  |  | * |
|  |  |  |  |  |  |  |
| treat*dia | 17°C-pH_T_7.9 | 17°C-pH_T_7.7 | 17°C-pH_T_7.4 | 21°C-pH_T_7.9 | 21°C-pH_T_7.7 | 21°C-pH_T_7.4 |
| 17°C-pH_T_7.9 |  | *** |  |  |  | ** |
| 17°C-pH_T_7.7 |  |  | *** | *** | *** | *** |
| 17°C-pH_T_7.4 |  |  |  |  |  | * |
| 21°C-pH_T_7.9 |  |  |  |  |  | * |
| 21°C-pH_T_7.7 |  |  |  |  |  | ** |
|  |  |  |  |  |  |  |
| treat*spine | 17°C-pH_T_7.9 | 17°C-pH_T_7.7 | 17°C-pH_T_7.4 | 21°C-pH_T_7.9 | 21°C-pH_T_7.7 | 21°C-pH_T_7.4 |
| 17°C-pH_T_7.9 |  | ** | * |  | *** | ** |
| 17°C-pH_T_7.7 |  |  |  | * | * |  |
| 17°C-pH_T_7.4 |  |  |  |  | *** | * |
| 21°C-pH_T_7.9 |  |  |  |  | *** | ** |
| 21°C-pH_T_7.7 |  |  |  |  |  |  |
|  |  |  |  |  |  |  |
| treat*hei | 17°C-pH_T_7.9 | 17°C-pH_T_7.7 | 17°C-pH_T_7.4 | 21°C-pH_T_7.9 | 21°C-pH_T_7.7 | 21°C-pH_T_7.4 |
| 17°C-pH_T_7.9 |  | *** | * | * | *** | * |
| 17°C-pH_T_7.7 |  |  | *** | *** | *** | *** |
| 17°C-pH_T_7.4 |  |  |  | * | * |  |
| 21°C-pH_T_7.9 |  |  |  |  | *** | * |
| 21°C-pH_T_7.7 |  |  |  |  |  |  |

**Table S2.** Details for Model2 concerning detachment velocity (VDet, Poisson distribution) variance of *Paracentrotus lividus* with treatment (treat) as fixed factor and behavioural variables as covariates: density of attached tube feet relative to oral test surface area (TFatt), active movement displacement (VMov) and direction (DirMov), spine angle (Spine°), circularity (a shape indices) and their interactions. Aquarium (replicate) was nested within treatment. P-values codes: ***: p≤0.001, **: 0.001<p≤0.01, *: 0.01<p≤0.05, *m: marginally significant difference.

|  | **Model 2: Generalized linear mixed-effect model** | | | | |  |
| --- | --- | --- | --- | --- | --- | --- |
| Fit by: | maximum likelihood (Laplace Approximation) | | | | | |
| Family: | poisson ( log ) | | | | | |
| Formula: | VDet ~ treat + + TFatt + Spine° + VMov + DirMov + Circularity + TFatt:Spine° + Spine°:Circularity + VMov:DirMov + DirMov:Circularity + (1\|aquarium) | | | | | |
|  |  | | | | |  |
|  | **AIC** | **BIC** | **logLik** | **deviance** | **df.resid** |  |
|  | 609.6 | 647.6 | -288.6 | 577.3 | 65 |  |
|  |  | | | | |  |
|  | **Scaled residuals:** | | | | |  |
|  | Min | 1Q | Median | 3Q | Max |  |
|  | -2.614 | -0.910 | 0.096 | 0.793 | 2.874 |  |
|  |  |  |  |  |  |  |
|  | **Random effects:** | | | | |  |
|  | Groups | Name | Variance | Std.Dev. |  |  |
|  | aquarium | (Intercept) | 0 | 0 |  |  |
|  | Number of obs: 81, groups: aquarium, 18 | | | | |  |
|  |  |  |  |  |  |  |
| **Fixed effects:** | | | | | | |
| **treatment** | 17°C-pH_T_7.9 | 17°C-pH_T_7.7 | 17°C-pH_T_7.4 | 21°C-pH_T_7.9 | 21°C-pH_T_7.7 | 21°C-pH_T_7.4 |
| 17°C-pH_T_7.9 |  |  |  |  | * |  |
| 17°C-pH_T_7.7 |  |  |  |  |  |  |
| 17°C-pH_T_7.4 |  |  |  |  | ** |  |
| 21°C-pH_T_7.9 |  |  |  |  | ** |  |
| 21°C-pH_T_7.7 |  |  |  |  |  | * |
|  |  |  |  |  |  |  |
|  | 17°C-pH_T_7.9 | 17°C-pH_T_7.7 | 17°C-pH_T_7.4 | 21°C-pH_T_7.9 | 21°C-pH_T_7.7 | 21°C-pH_T_7.4 |
| Spine° | *** | *** | *** | *** | *** | *** |
| TFatt | ** | ** | ** | ** | ** | ** |
| VMov | *** | *** | *** | *** | *** | *** |
| DirMov | ** | ** | ** | ** | ** | ** |
| Circularity | * | * | * | ** | ** | * |
|  |  |  |  |  |  |  |
|  | 17°C-pH_T_7.9 | 17°C-pH_T_7.7 | 17°C-pH_T_7.4 | 21°C-pH_T_7.9 | 21°C-pH_T_7.7 | 21°C-pH_T_7.4 |
| TFatt:Spine° |  |  |  |  |  |  |
| Spine°:Circularity | ** | ** | ** | ** | ** | ** |
| VMov:DirMov | *m | *m | *m | *m | *m | *m |
| DirMov:Circularity | * | * | * | * | * | * |

**Table S3.** Details for Model3 concerning active movement velocity (VMov, negative binomial distribution) of *Paracentrotus lividus* according to treatment (treat), flow velocity (vf) and their interaction (treat*vf), with aquarium (replica) being nested within treatment. For each fixed effect, significant p-values between treatments are shown. In brackets is indicated whether the effect of treat*vf is positive or negative. P-values codes: ***: p≤0.001, **: 0.001<p≤0.01, *: 0.01<p≤0.05, *m: marginally significant difference.

|  | **Model 3: Generalized linear mixed-effect model** | | | | |  |
| --- | --- | --- | --- | --- | --- | --- |
| Fit by: | maximum likelihood (Laplace Approximation) | | | | | |
| Family: | negative binomial ( log ) | | | | | |
| Formula: | VMov ~ treat + vf + treat:vf + (1\|aquarium) | | | | | |
|  |  | | | | | |
|  | **AIC** | **BIC** | **logLik** | **deviance** | **df.resid** |  |
|  | 6219.3 | 6284.3 | -3095.7 | 6191.3 | 754 |  |
|  |  | | | | |  |
|  | **Scaled residuals:** | | | | |  |
|  | Min | 1Q | Median | 3Q | Max |  |
|  | -0.5989 | -0.5728 | --0.2775 | 0.1482 | 1.0546 |  |
|  |  |  |  |  |  |  |
|  | **Random effects:** | | | | |  |
|  | Groups | Name | Variance | Std.Dev. |  |  |
|  | aquarium | (Intercept) | 0.05573 | 0.2361 |  |  |
|  | Number of obs: 768, groups: aquarium, 18 | | | | |  |
|  |  |  |  |  |  |  |
| **Fixed effects:** | | | | | | |
| **treat** | 17°C-pH_T_7.9 | 17°C-pH_T_7.7 | 17°C-pH_T_7.4 | 21°C-pH_T_7.9 | 21°C-pH_T_7.7 | 21°C-pH_T_7.4 |
| 17°C-pH_T_7.9 |  | *** |  | *** |  |  |
| 17°C-pH_T_7.7 |  |  |  |  |  | *** |
| 17°C-pH_T_7.4 |  |  |  | *** |  | ** |
| 21°C-pH_T_7.9 |  |  |  |  |  | *** |
| 21°C-pH_T_7.7 |  |  |  |  |  | *** |
|  |  |  |  |  |  |  |
|  | 17°C-pH_T_7.9 | 17°C-pH_T_7.7 | 17°C-pH_T_7.4 | 21°C-pH_T_7.9 | 21°C-pH_T_7.7 | 21°C-pH_T_7.4 |
| **vf** | *** | *** | *** | *** | *** | *** |
|  |  |  |  |  |  |  |
| **treat*vf** | 17°C-pH_T_7.9 | 17°C-pH_T_7.7 | 17°C-pH_T_7.4 | 21°C-pH_T_7.9 | 21°C-pH_T_7.7 | 21°C-pH_T_7.4 |
| 17°C-pH_T_7.9 |  | (+) *** |  | (+) *** | (+) * |  |
| 17°C-pH_T_7.7 |  |  | (-)*m |  |  | (-) *** |
| 17°C-pH_T_7.4 |  |  |  | (+) *** |  | (-) *** |
| 21°C-pH_T_7.9 |  |  |  |  |  | (-) *** |
| 21°C-pH_T_7.7 |  |  |  |  |  | (-) *** |

**S5. Results concerning microhabitat conditions.**

Coastal seawater pH_T-SW_ was 8.10±0.05 (n=4). In the big pool (B-pool), pH_T-SW_ varied with time (F_(3,44)_=105.8, p<0.001) and compartment (below and over, F_(1,44)_=41.6, p<0.001) and marginally with their interaction (F_(3,44)_=2.908, p=0.045, n=12). pH_T-SW_ below and over the sea urchins showed the highest values after day-LT (below:8.42±0.13, over:8.57±0.11) and the lowest (below:7.68±0.06, over:7.72±0.11) after night-LT (Fig S2). This pattern was consistent with single measurements of pool water column (pH_T-SW-after_day-LT_=8.82, pH_T-SW-after_night-LT_=7.66). pH_T-SW_ below the sea urchins was significantly lower than that over them, before (p≤0.001) and after (p=0.013) the day-LTs, but not during the night-LTs. Coelomic fluid pH_T-CF_ ranged between 7.31 to 7.92.

Seawater A_T-SW_ was not affected by time (F_3,37_=0.33, p=0.81) nor by compartment (F_(1,39)_=0.860, p=0.36). A_T-SW_ below the sea urchins ranged from 1.94 to 3.48 mmol kg^-1^ while that over them ranged from 1.89 to 3.14 mmol kg^-1^. Single A_T-SW_ samples of the pool water column during a nychthemeral cycle ranged from 2.12 to 2.47 mmol kg^-1^.

The comparisons performed only after the night-LT revealed that pH_T-SW_ significantly varied between pools (F_(1,22)_=37.8, p≤0.001) and compartments (F_(1,22)_=5.01, p=0.036) with no interactive effect. A_T-SW_ did not differ with any factor. pH_T-SW_ below (7.52±0.07, n=12) and over (7.54±0.05, n=12) the sea urchins was significantly lower in the small pool (S-pool) than in B-pool (Fig S2).


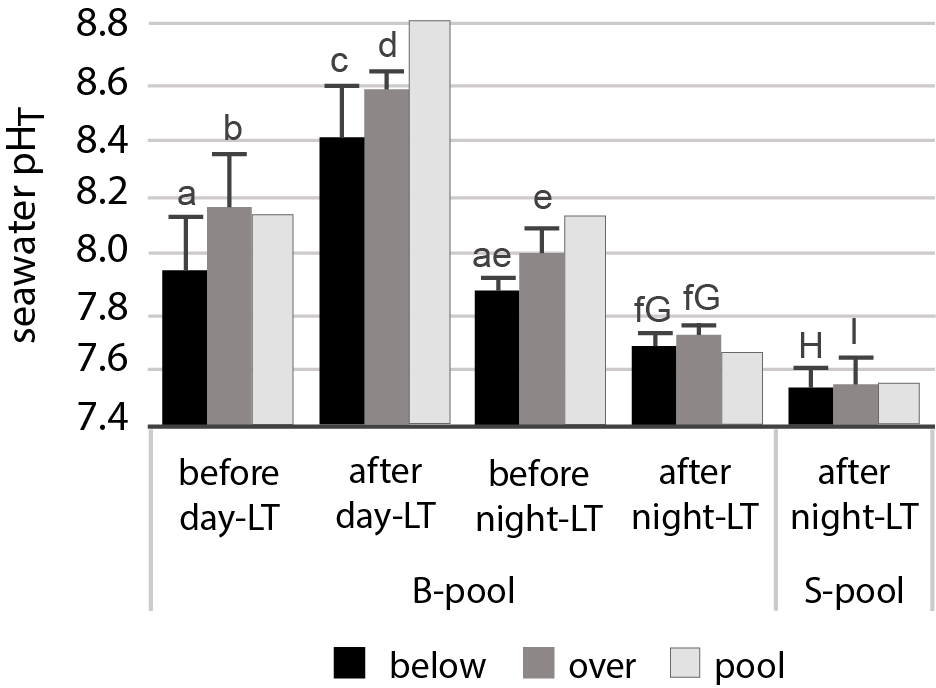


**Figure S2.** Seawater pH_T_ (total scale) measured within sea urchin self-burrowed pits (n=12) of two intertidal pools (B-pool, S-pool). pH_T-SW_ was measured at 4 periods of the nychthemeral cycle: 2h before and 2h after the low tide (LT) during both day and night tides for B-pool, while only 2h after the night tide for S-pool. During each period, pH_T_ was measured below and over the same sea urchin (n=12). Light grey bars indicate single measurements of pH_T-SW_ per time. Significant differences between compartments and times in the B-pool are indicated by lowercase letters; while between compartments and pools after the night-LT by uppercase letters; means sharing the same letter are not significantly different (pTukey≥0.05).

**Table S4.** Adhesive measurements (mean±sd, n=3) of whole individuals and tube foot disk in *Paracentrotus lividus* according to experimental treatment taken at two times (w1: week1 and w8: week8). ANOVA results showing F statistic and p-values. Significant differences between means of treatments are indicated by letters in superscript; means sharing the same superscript are not significantly different (pTukey ≥ 0.05).

|  |  | **Treatments** | | | | | | **ANOVA** | |
| --- | --- | --- | --- | --- | --- | --- | --- | --- | --- |
|  | **time** | **17°C-pH_T_7.9** | **17°C-pH_T_7.7** | **17°C-pH_T_7.4** | **21°C-pH_T_7.9** | **21°C-pH_T_7.7** | **21°C-pH_T_7.4** | **F_(5,12)_** | **p-value** |
| **Sea urchin detachment force (F_urchin_, N)** | w1 | 1.51±0.01^ab^ | 0.62±0.29 ^b^ | 0.72±0.28 ^b^ | 1.70±0.29 ^a^ | 0.80±0.22^ab^ | 1.28±0.67^ab^ | 4.662 | 0.013 |
|  | w8 | 0.26±0.16 | 0.37±0.17 | 0.49±0.33 | 0.37±0.28 | 0.39±0.22 | 0.33±0.01 | 0.601 | 0.7 |
| **Disk tenacity**  **(T_disk_, MPa)** | w1 | 0.11±0.03 | 0.08±0.03 | 0.10±0.05 | 0.20±0.07 | 0.17±0.05 | 0.17±0.05 | 2.196 | 0.123 |
|  | w8 | 0.05±0.03 | 0.08±0.03 | 0.10±0.08 | 0.10±0.06 | 0.08±0.01 | 0.13±0.01 | 0.862 | 0.536 |
| **Disk detachment force (F_disk_, N)** | w1 | 0.03±0.01 | 0.02±0.01 | 0.03±0.01 | 0.05±0.02 | 0.04±0.02 | 0.04±0.01 | 1.966 | 0.156 |
|  | w8 | 0.02±0.01 | 0.02±0.01 | 0.03±0.02 | 0.02±0.01 | 0.02±0.00 | 0.03±0.00 | 0.524 | 0.754 |
| **Disk adhesive surface (S_disk_, mm²)** | w1 | 0.26±0.03 | 0.29±0.02 | 0.28±0.01 | 0.27±0.02 | 0.27±0.02 | 0.27±0.01 | 0.805 | 0.567 |
|  | w8 | 0.24±0.01 | 0.26±0.01 | 0.26±0.03 | 0.23±0.01 | 0.26±0.03 | 0.27±0.02 | 1.771 | 0.193 |

**Table S5.** ANOVA results for cross-sectional surface area of the stem connective tissue layer and stem mechanical properties at week1 and 8 (w1, w8).

|  | **w1** | | **w8** | |
| --- | --- | --- | --- | --- |
|  | **F_(5,12)_** | **p-value** | **F_(5,12)_** | **p-value** |
| **Breaking Force** | 4.29 | 0.018 | 4.18 | 0.020 |
| **Cross-sectional surface area of the stem connective tissue layer** | 16.83 | <0.001 | 2.05 | 0.142 |
| **Extensibility** | 2.11 | 0.134 | 1.89 | 0.169 |
| **Tensile strength** | 8.21 | 0.001 | 6.05 | 0.005 |
| **Stiffness** | 7.85 | 0.002 | 4.43 | 0.016 |
| **Toughness** | 5.08 | 0.010 | 4.10 | 0.021 |

**
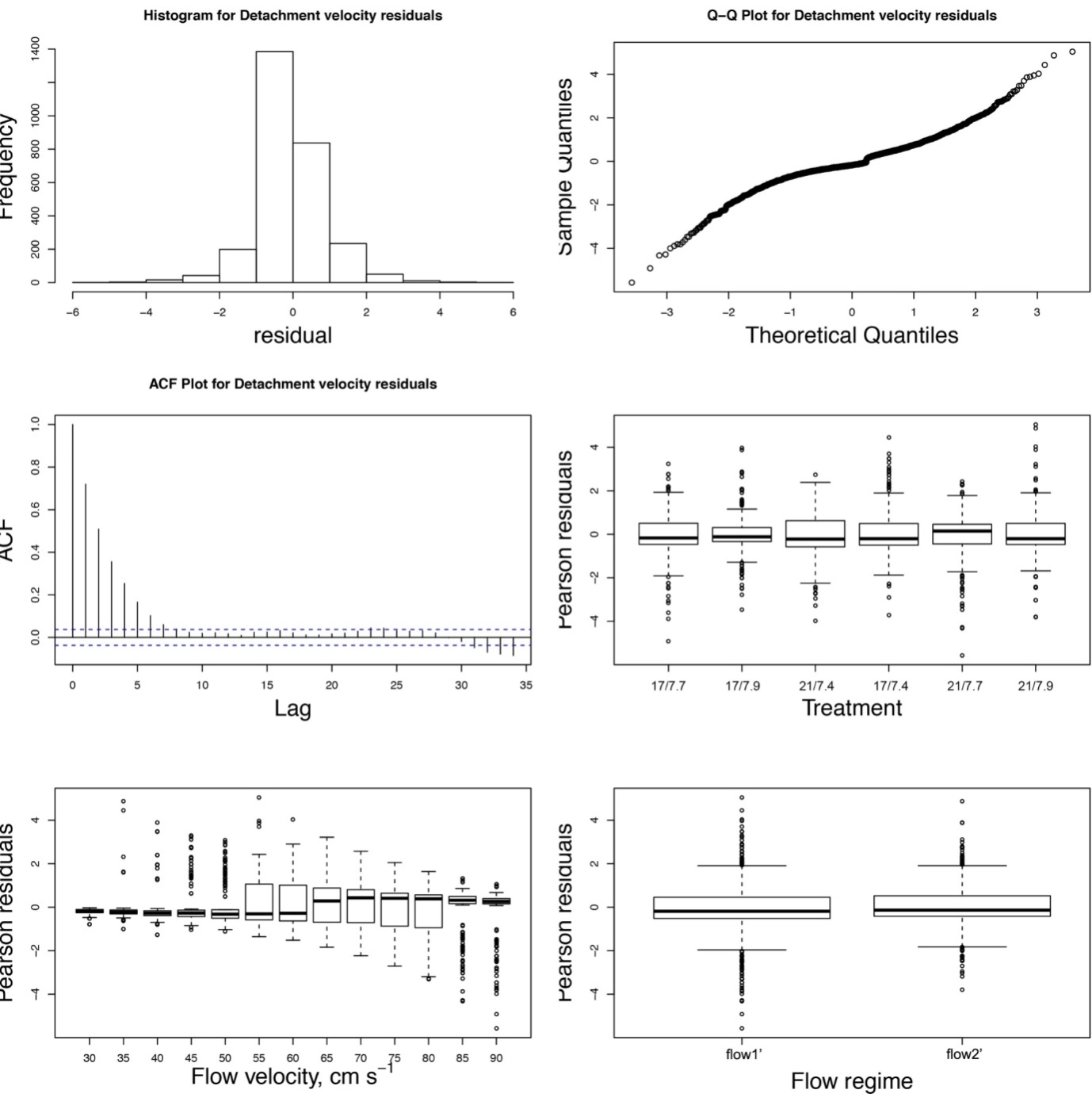
**

**Figure S4.** Model1 validation concerning the probability of dislodgement of *Paracentrotus lividus*. It is shown the covariates plotted against their own standardized Pearson’s residuals as well as and residuals for probability of detachment plotted for normality and for auto and cross-covariance and correlation (ACF).

**
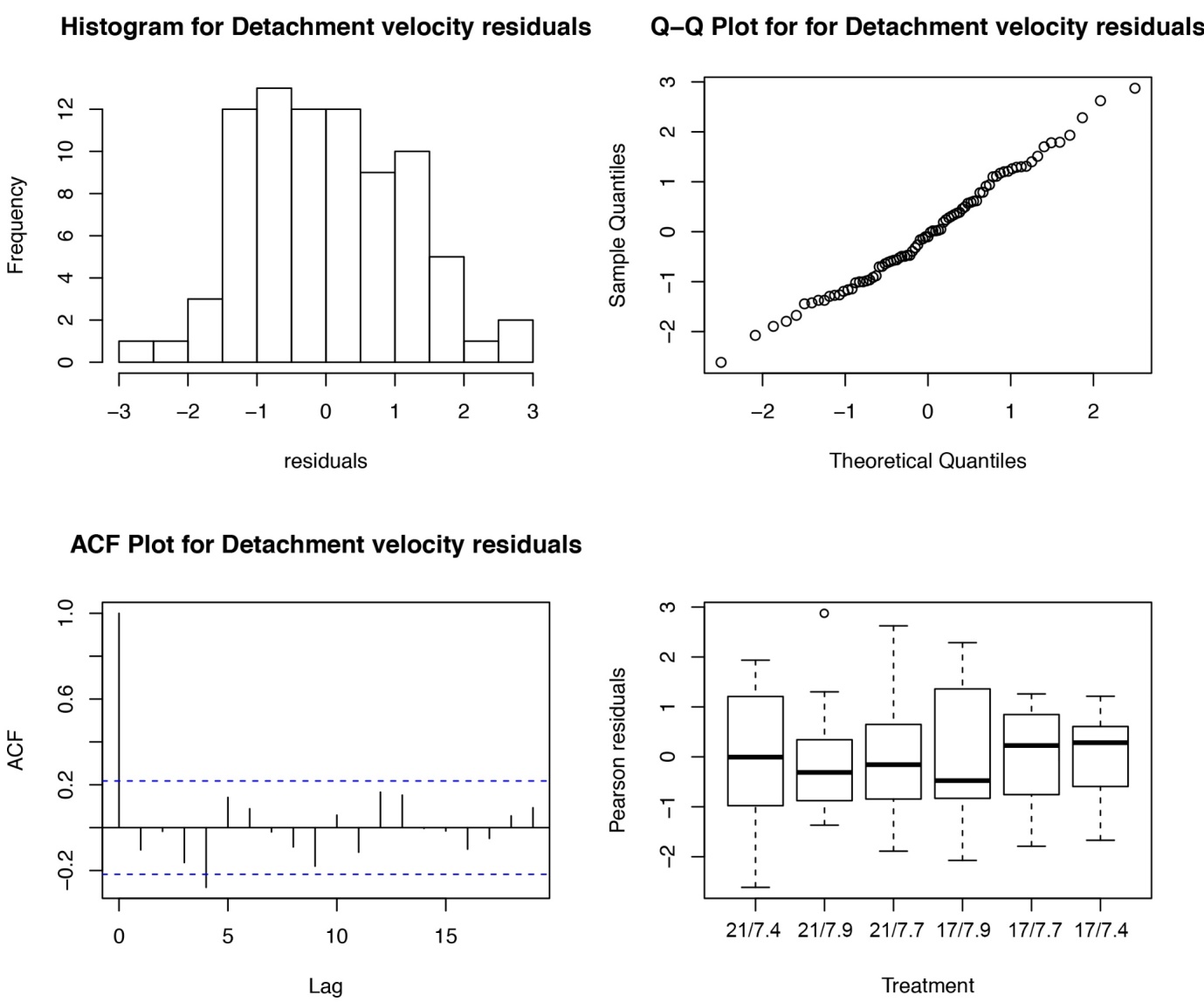
**

**Figure S5.** Model2 validation concerning detachment velocity (VDet) variance of *Paracentrotus lividus.* It is shown the covariates plotted against their own standardized Pearson’s residuals as well as and detachment velocity residuals plotted for normality and for auto and cross-covariance and correlation (ACF).

**
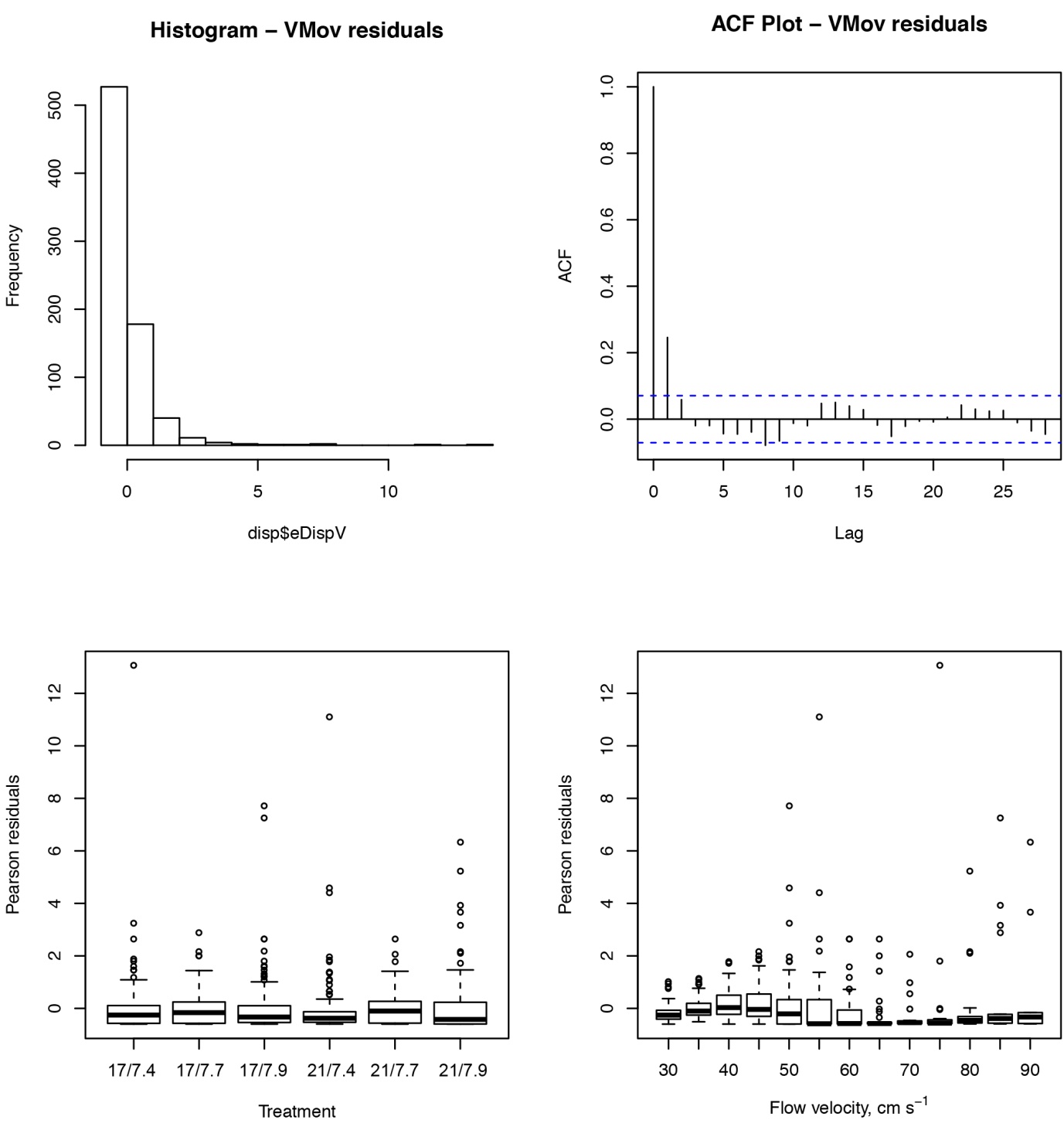
Figure S6.** Model3 validation concerning active movement velocity (VMov) of *Paracentrotus lividus*.


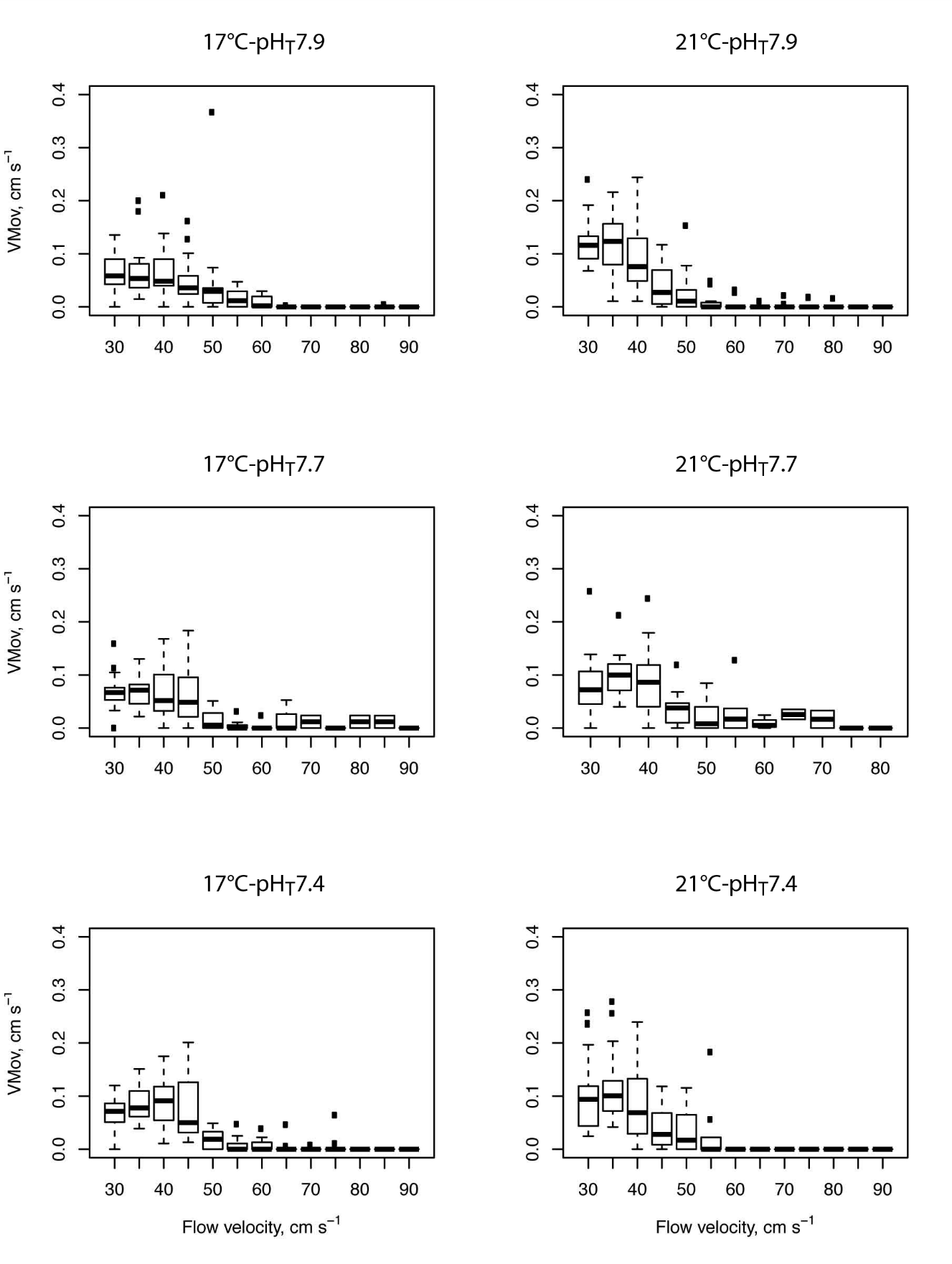


**Figure S7.** Boxplots for active movement velocity (V_Mov_, in cm s-1) per flow velocity and treatment. Boxes include 50% of the observations with a black thick horizontal line representing median values, and lower and upper limits delineating 25% and 75% quartiles, which is referred as the interquartile range. Upper and lower whiskers denote minimum and maximum observations outside the interquartile range and excluding outliers. Outliers represented by individual points.

**Figure S8.** Proportion of individuals moving upstream or downstream according to flow velocity (VF, cm s^-1^).

**Table S6.** Density of total attached tube feet relative to oral test surface (TF_att_, in mm^-2^) and percentage of total attached tube feet relative to the number of adoral tube feet (TF_att_%, in %) per flow velocity (V_F_) for each treatment. In brackets is noted the percentage of detached tube feet per V_F_ in respect to the maximal percentage of attached tube feet per treatment. Densities and percentages are given as mean±sd, with n=18 at V_F_30 for every treatment, then it decreases with V_F_ as the urchins detached in each treatment. TFad: adoral tube feet.

| **TF_att_** | | | | | | |
| --- | --- | --- | --- | --- | --- | --- |
| **V_F_** | **17°C-pH_T_7.9** | **17°C-pH_T_7.7** | **17°C-pH_T_7.4** | **21°C-pH_T_7.9** | **21°C-pH_T_7.7** | **21°C-pH_T_7.4** |
| **30** | 0.113±0.050 | 0.149±0.061 | 0.120±0.042 | 0.146±0.056 | 0.103±0.040 | 0.163±0.045 |
| **35** | 0.144±0.047 | 0.143±0.056 | 0.115±0.036 | 0.142±0.042 | 0.102±0.035 | 0.157±0.079 |
| **40** | 0.136±0.047 | 0.137±0.057 | 0.114±0.040 | 0.127±0.050 | 0.098±0.036 | 0.152±0.100 |
| **45** | 0.132±0.049 | 0.125±0.063 | 0.113±0.037 | 0.128±0.050 | 0.092±0.041 | 0.131±0.102 |
| **50** | 0.115±0.043 | 0.126±0.073 | 0.119±0.047 | 0.105±0.049 | 0.082±0.033 | 0.126±0.120 |
| **55** | 0.104±0.047 | 0.115±0.058 | 0.115±0.036 | 0.100±0.049 | 0.062±0.036 | 0.140±0.131 |
| **60** | 0.092±0.039 | 0.097±0.056 | 0.101±0.059 | 0.101±0.049 | 0.072±0.021 | 0.126±0.128 |
| **65** | 0.078±0.029 | 0.077±0.061 | 0.093±0.046 | 0.093±0.054 | 0.062±0.019 | 0.140±0.111 |
| **70** | 0.064±0.028 | 0.067±0.059 | 0.051±0.028 | 0.101±0.064 | 0.053±0.014 | 0.087±0.062 |
| **75** | 0.046±0.022 | 0.071±0.051 | 0.075±0.064 | 0.097±0.050 | 0.046±0.025 | 0.066±0.025 |
| **80** | 0.039±0.009 | 0.074±0.078 | 0.066±0.071 | 0.087±0.013 | 0.043±0.027 | 0.061±0.034 |
| **85** | 0.030±0.013 | 0.060±0.055 | 0.069±0.052 | 0.092±0.031 | 0.039±0.021 | 0.068±0.057 |
| **90** | 0.027±0.013 | 0.071±0.087 | 0.065±0.046 | 0.078±0.034 | 0.041±0.031 | 0.059±0.034 |
|  |  |  |  |  |  |  |
| **TF_att_%** | | | | | | |
| **V_F_** | **17°C-pH_T_7.9** | **17°C-pH_T_7.7** | **17°C-pH_T_7.4** | **21°C-pH_T_7.9** | **21°C-pH_T_7.7** | **21°C-pH_T_7.4** |
| **30** | 14.9±7.0 (-21%) | 18.6±5.6 (0%) | 15.8±4.7 (0%) | 18.1±6.8 (0%) | 12.6±4.0 (0%) | 18.4±4.8 (0%) |
| **35** | 18.8±5.4 (0%) | 17.9±5.2 (-4%) | 15.0±4.8 (-5%) | 17.7±6.0 (-2%) | 12.6±3.4 (0%) | 17.5±8.1 (-5%) |
| **40** | 17.8±5.7 (-6%) | 17.2±5.3 (-8%) | 15.1±5.7 (-4%) | 15.7±7.0 (-13%) | 12.3±4.2 (-2%) | 16.7±10.0 (-9%) |
| **45** | 17.1±6.1 (-9%) | 15.5±6.3 (-17%) | 15.2±6.1 (-4%) | 15.9±6.8 (-12%) | 11.4±4.6 (-10%) | 14.7±10.5 (-20%) |
| **50** | 15.2±6.4 (-19%) | 15.4±7.3 (-17%) | 16.8±7.4 (6%) | 13.3±7.2 (-26%) | 10.2±3.5 (-19%) | 14.1±12.5 (-23%) |
| **55** | 13.9±6.9 (-26%) | 14.2±5.4 (-24%) | 15.7±5.4 (-1%) | 13.3±7.1 (-26%) | 7.5±3.6 (-40%) | 16.1±13.4 (-12%) |
| **60** | 12.3±5.2 (-35%) | 11.9±5.6 (-36%) | 13.9±8.8 (-12%) | 13.3±7.4 (-26%) | 9.0±1.3 (-28%) | 14.3±13.0 (-22%) |
| **65** | 10.3±3.7 (-45%) | 9.3±6.2 (-50%) | 12.5±6.9 (-21%) | 11.9±7.2 (-34%) | 7.2±2.0 (-43%) | 16.0±10.4 (-13%) |
| **70** | 8.4±3.5 (-55%) | 8.2±5.9 (-56%) | 6.9±3.7 (-57%) | 13.2±8.3 (-27%) | 6.3±2.1 (-50%) | 10.5±5.5 (-43%) |
| **75** | 5.9±2.7 (-69%) | 8.7±5.0 (-53%) | 10.4±10.2 (-34%) | 12.5±7.0 (-31%) | 4.9±1.1 (-61%) | 8.2±3.4 (-56%) |
| **80** | 4.9±1.8 (-74%) | 8.7±7.8 (-53%) | 9.2±10.9 (-41%) | 11.4±3.0 (-37%) | 4.6±1.4 (-64%) | 7.4±3.6 (-59%) |
| **85** | 3.6±1.5 (-81%) | 7.1±5.4 (-62%) | 9.7±8.5 (-39%) | 12.7±4.3 (-29%) | 4.2±0.9 (-67%) | 8.0±5.6 (-56%) |
| **90** | 3.2±1.7 (-83%) | 8.1±8.8 (-56%) | 9.1±7.6 (-42%) | 10.8±4.7 (-40%) | 4.0±1.7 (-68%) | 5.6±3.9 (-69%) |
| **TFad** | 653.7±114.2 | 466.7±38.0 | 460.6±28.6 | 472.2±34.9 | 489.4±66.3 | 476.7±55.4 |


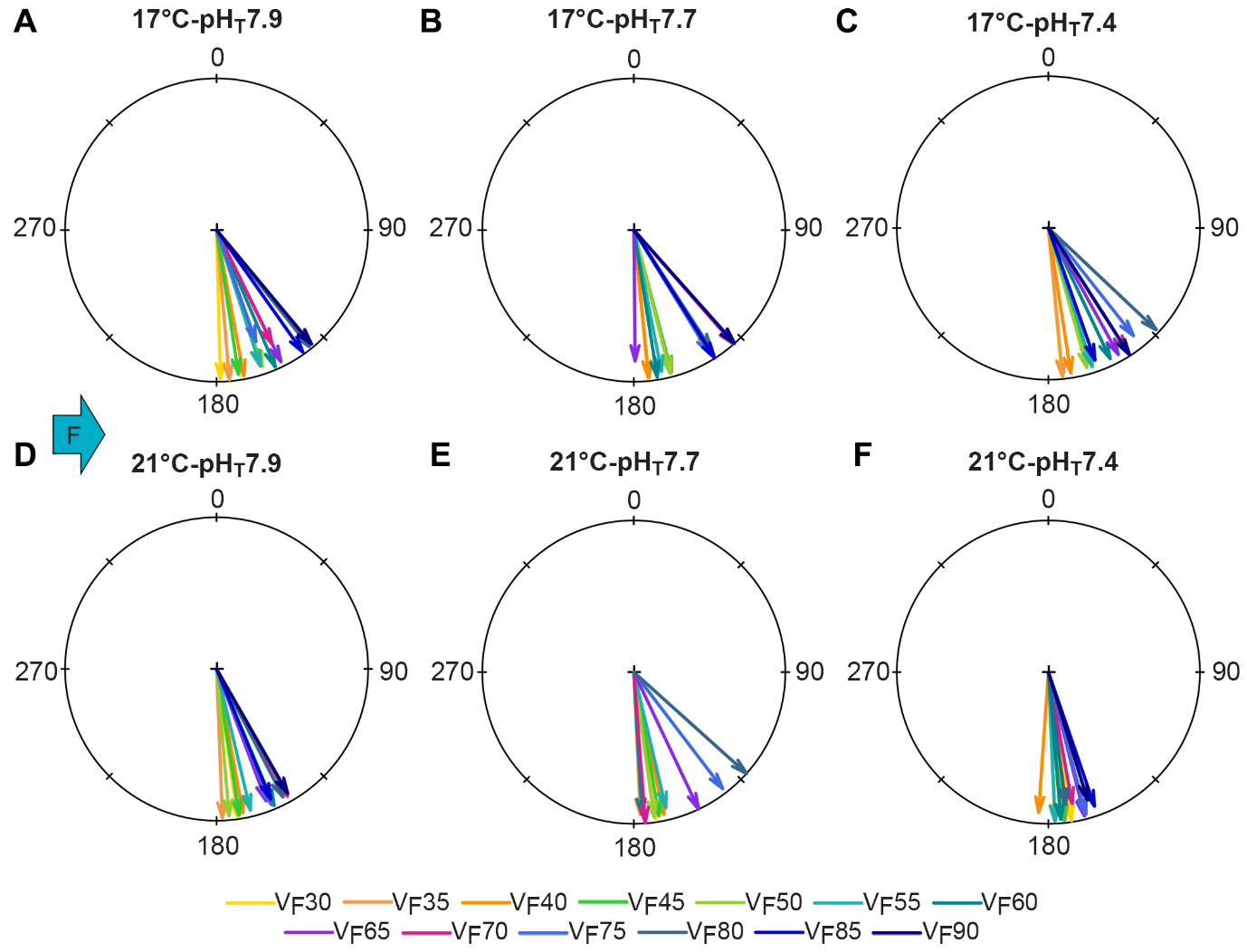


**Figure S10.** Mean vectors of planform spine angle (in degrees) per flow velocity (V_F_) and treatment of *Paracentrotus lividus* measured in the zone of the urchin comprised between 170° and 190°. Vector length is inversely proportional to data dispersion. Blue arrow showing flow provenance (F), with angles between 0-180° and 180-359° implying spines orientated downstream and upstream, respectively.

**Supplementary references**

Agüera A, Brophy D (2011) Use of saggital otolith shape analysis to discriminate Northeast Atlantic and Western Mediterranean stocks of Atlantic saury, *Scomberesox saurus* *saurus* (Walbaum). *Fisheries Research*, **110**, 465–471.

Batschelet E (1981) *Circular statistics in biology*. Academic Press, London, New York.

Benjamini Y, Hochberg Y (1995) Controlling the false discovery rate: A practical and powerful approach to multiple testing. *Journal of the Royal Statistical Society. Series B (Methodological)*, **57**, 289–300.

Claessens T (2009) Capacités d’acclimatation de l’oursin comestible (*Paracentrotus lividus*) à l’acidification des océans : étude *in-situ* en mares intertidales. Master Thesis, Université Libre de Bruxelles

Collard M, Laitat K, Moulin L, Catarino AI, Grosjean P, Dubois P (2013) Buffer capacity of the coelomic fluid in echinoderms. *Comparative biochemistry and physiology. Part A, Molecular & integrative physiology*, **166**, 199–206.

Doncaster CP, Davey AJH (2007) *Analysis of variance and covariance: how to choose and construct models for the life sciences*. Cambridge University, Cambridge.

Ebert TA (1980) Relative Growth of Sea Urchin Jaws : an Example of Plastic Resource Allocation. *Bull Marine Science*, **30**, 467–474.

Ebert TA, Hernandez JC, Clemente S (2014) Annual reversible plasticity of feeding structures: cyclical changes of jaw allometry in a sea urchin. *Proceedings of the Royal Society B: Biological Sciences*, **281**, 20132284–20132284.

Edwards PB, Ebert TA (1991) Plastic responses to limited food availability and spine damage in the sea urchin *Strongylocentrotus purpuratus* (Stimpson). *Journal of Experimental Marine Biology and Ecology*, **145**, 205–220.

Gran G (1950) Determination of the equivalent point in potentiometric titrations. *Acta Chemica Scandinavica*, **4**, 559–577.

Moulin L, Catarino AI, Claessens T, Dubois P (2011) Effects of seawater acidification on early development of the intertidal sea urchin *Paracentrotus lividus* (Lamarck 1816). *Marine Pollution Bulletin*, **62**, 48–54.

Pewsey A, Neuhäuser M, Ruxton GD (2013) *Circular statistics in R*. Oxford University Press, Oxford.

Zar JH (1999) *Biostatistical analysis*. Prentice Hall, New Jersey.
