# Supplementary material for: Ocean warming and acidification alter the behavioral response to flow of the sea urchin Paracentrotus lividus
Source: Ecol Evol. 2019 Oct 17;9(21):12128–43. doi: 10.1002/ece3.5678 (PMC6854335; doi:10.1002/ece3.5678)
Supplement: Supplementary file 2 [file ECE3-9-12128-s002.docx]

**List of abbreviations**

| **Abbreviations** | **Parameters** |
| --- | --- |
| A_T-SW_/A_T-CF_ | total alkalinity of the seawater/coelomic fluid (mmol kg_SW_^-1^) |
| CF | coelomic fluid |
| Dir_Mov_ | sea urchin active movement (degrees) |
| d_test_ | ambital test diameter (mm) |
| d_urchin_ | ambital sea urchin diameter with spines (mm) |
| F_disk_ | disk detachment force (Newton) |
| F_urchin_ | sea urchin detachment force (Newton) |
| Flow1’/ Flow2’ | flow regime with flow velocity increments every minute/two minutes |
| h_test_ | test height (mm) |
| l_spine_ | ambital spine length (mm) |
| pH_Nist/T_ | pH in NIST/total scale (pH units) |
| S_CT_ | cross-sectional surface area of the stem connective tissue layer |
| S_disk_ | tube foot disk adhesive surface area (mm^2^) |
| S_urchin_ | sea urchin adhesive surface area (mm^2^) |
| Spine° | spine angle (degrees) |
| T_disk_ | disk tenacity (MPa) |
| TF | tube feet |
| T_urchin_ | sea urchin tenacity (MPa) |
| TF_att_ | density of attached tube feet relative to oral test surface area (mm^-2^) |
| TF_att_% | percentage of attached tube feet relative to the number adoral tube feet (%) |
| V_Det_ | detachment velocity (cm s^-1^) |
| V_F_ | flow velocity (cm s^-1^) |
| V_Mov_ | active movement velocity (cm s^-1^) |
| w1/8/12 | week 1/8/12 |
